# Identification of novel prostate cancer drivers using RegNetDriver: a framework for integration of genetic and epigenetic alterations with tissue-specific regulatory network

**DOI:** 10.1186/s13059-017-1266-3

**Published:** 2017-07-27

**Authors:** Priyanka Dhingra, Alexander Martinez-Fundichely, Adeline Berger, Franklin W. Huang, Andre Neil Forbes, Eric Minwei Liu, Deli Liu, Andrea Sboner, Pablo Tamayo, David S. Rickman, Mark A. Rubin, Ekta Khurana

**Affiliations:** 1 Department of Physiology and Biophysics, Weill Cornell Medical College, New York, New York 10065 USA; 2 Institute for Computational Biomedicine, Weill Cornell Medical College, New York, New York 10021 USA; 3 Department of Pathology and Laboratory Medicine, Weill Cornell Medical College, New York, New York 10065 USA; 4 Department of Medical Oncology, Dana-Farber Cancer Institute, 450 Brookline Avenue, Boston, MA 02215 USA; 5 Department of Medicine, Harvard Medical School, 25 Shattuck Street, Boston, MA 02115 USA; 6 Cancer Program, The Broad Institute of Harvard and MIT, 415 Main Street, Cambridge, MA 02142 USA; 7 Department of Urology, Weill Cornell Medical College, New York, New York 10065 USA; 8 Caryl and Israel Englander Institute for Precision Medicine, New York Presbyterian Hospital-Weill Cornell Medicine, New York, NY 10065 USA; 9 Department of Medicine, University of California San Diego, La Jolla, California USA; 10 Moores Cancer Center, University of California San Diego, La Jolla, California USA; 11 Meyer Cancer Center, Weill Cornell Medical College, New York, New York 10065 USA

**Keywords:** Tissue-specific regulatory network, Cancer drivers, Single nucleotide variants, Structural variants, DNA methylation, Prostate cancer

## Abstract

We report a novel computational method, RegNetDriver, to identify tumorigenic drivers using the combined effects of coding and non-coding single nucleotide variants, structural variants, and DNA methylation changes in the DNase I hypersensitivity based regulatory network. Integration of multi-omics data from 521 prostate tumor samples indicated a stronger regulatory impact of structural variants, as they affect more transcription factor hubs in the tissue-specific network. Moreover, crosstalk between transcription factor hub expression modulated by structural variants and methylation levels likely leads to the differential expression of target genes. We report known prostate tumor regulatory drivers and nominate novel transcription factors (ERF, CREB3L1, and POU2F2), which are supported by functional validation.

**Electronic supplementary material:**

The online version of this article (doi:10.1186/s13059-017-1266-3) contains supplementary material, which is available to authorized users.

## Background

Cancer is a disease of the genome, characterized by uncontrolled growth and survival of damaged cells [1]. Prostate cancer (PCa) is the second most common cancer in men worldwide [2]. Whole-exome sequencing and whole-genome sequencing (WGS) of tumors has revealed recurrent genomic alterations in PCa [3–8]. Genomic alterations range from single nucleotide variants (SNVs) to large structural variants (SVs) [5, 9, 10]. SVs include deletions, insertions, duplications, inversions, translocations, and other complex rearrangements. The most common genomic alteration identified in prostate tumors is the fusion of the 5′ untranslated region of *TMPRSS2* with *ERG* caused by deletions or translocations, which is found in 40–50% of samples [3, 11, 12]. Other frequent alterations include: chromosomal deletions involving loss of *NKX3.1* [13, 14], *PTEN* [15–17], *TP53*, *CHD1*, or *CDKN1B* [18]; genomic gains of chr 7 and 8q; and focal amplifications of *MYC*, *PIK3CA*, *FGFR1*, and *WHSC1L1* [3]. Additionally, recurrent SNVs have been identified in *SPOP*, *FOXA1*, *TP53*, *MED12*, *IDH1*, and *PTEN* [3, 5–8]. These known genomic alterations affect the genes involved in prostate development, cell cycle signaling, chromatin modification, androgen signaling, and many other processes [4]. Further, these alterations lead to substantial heterogeneity in tumor samples and have been used to define PCa molecular subtypes based on fusion of *ETS* family genes (*ERG*, *ETV*, *ETV4*, or *FLI1*) and mutations in *SPOP*, *FOXA1*, or *IDH1* [3].

Other than genomic changes, epigenetic alterations such as changes in DNA methylation, histone modifications, and chromatin organization (e.g., nucleosome remodeling and chromatin looping) impact gene expression and play an important role in the onset and progression of PCa [19–21]. Among the different epigenetic changes, DNA methylation is the most common and best characterized in PCa [3, 22–24]. Aberrant DNA methylation (hyper- or hypo-methylation) at promoter regions in PCa has been reported to be associated with gene expression changes [25]. For example, down-regulation of *GSTP1* activity in PCa due to DNA hyper-methylation in the promoter region has been associated with prostate carcinogenesis [26]. Other genes that commonly exhibit hyper-methylated promoters and have known implications in prostate tumorigenesis include *MGMT*, *CDKN2A*, *APC*, *AR*, and *ER* [22, 27]. Similarly, the high expression of *PLAU* and *CAGE* genes in prostate cells due to promoter hypo-methylation [23] has been associated with increased tumor invasion and metastasis [22].

Although the individual lists of genetic and epigenetic alterations have greatly enhanced our understanding of prostate tumorigenesis, these events do not act in isolation and it is important to interpret their integrative global effects on differential gene expression in cancer. This is because various events can alter the expression of a gene (1) SNVs or SVs in (a) the coding sequence of the gene, (b) the transcription factors (TFs) that regulate it, or (c) the associated non-coding regulatory regions (promoters and enhancers) or (2) epigenetic changes at the promoters and enhancers. Previous studies have focused on the identification of individual categories of alterations that occur more than expected randomly, and thus likely constitute drivers of tumorigenesis – for example, SNVs [3, 6, 9, 28] or SVs [3, 11, 29]. However, these alterations act in concert to influence tumor growth and it is important to integrate the different categories of alterations to identify the top candidates that are likely to play a major role in tumorigenesis by dysregulating thousands of genes, termed regulatory drivers. Here, we report a novel computational approach that makes use of tissue-specific regulatory networks to understand the global impact of genetic and epigenetic alterations affecting both coding and non-coding cis-regulatory regions (promoters and enhancers) and identify the regulatory drivers of tumorigenesis. Tissue-specific regulatory networks capture the molecular basis of gene regulation at a systems level and offer a unique means to understand the functional impact of genetic and epigenetic changes in TFs, their target genes, and non-coding cis-regulatory regions [30–32].

Regulatory networks are usually constructed using data from chromatin immunoprecipitation sequencing (ChIP-Seq) or gene co-expression assays. The networks derived from ChIP-Seq data are limited by the availability of antibodies corresponding to TFs and the difficulty of interrogating multiple TFs in a tissue-specific manner. The largest human regulatory network constructed using ENCODE ChIP-Seq data consists of only 119 TF genes, is not specific for prostate tissue, and does not contain important PCa TFs, such as *ERG* and *AR* [30]. While regulatory networks based on gene co-expression overcome the problem of studying one TF at a time, they suffer from the limitation that network edges, which represent significant co-expression relationships between genes (nodes), often correspond to protein–protein interactions as opposed to regulatory interactions [33]. Networks can be based on an alternative statistical measure, namely mutual information to detect dependence between every pair of genes from RNA expression [34–37]. However, the edges in both co-expression networks and those based on mutual information lack edge directionality (i.e., which TF gene is the regulator and which gene is regulated) and do not allow incorporation of cis-regulatory regions in the network. Overcoming these limitations, we report the use of DNase I hypersensitive sites (DHS) corresponding to accessible regulatory DNA regions to study directed tissue-specific regulatory interactions [38, 39]. Unlike previous studies, our approach for DHS-based network construction is not restricted to TF–TF interactions [32], rather we model the interactions of TFs with both TF and non-TF target genes and incorporate regulatory regions (promoters and enhancers) assisting those interactions.

We combined DHS data from prostate epithelial cells with other functional genomics data from ENCODE and the Roadmap Epigenomics Mapping Consortium (REMC) [40] to construct a comprehensive prostate regulatory network [40, 41]. Previous studies have shown that genes encoding TFs often play an important role in tumorigenesis [42–45]. Targeting TFs that regulate a large number of genes (TF hubs) and act as drivers of cellular transformation from normal to malignant state can offer novel therapeutic options [42]. While *ERG* and *AR* are well known TF genes that play an important role in prostate tumorigenesis, a recent study has also reported *FOXM1* and a non-TF gene *CENPF* as master regulators of PCa malignancy [46]. However, a comprehensive analysis connecting the tumorigenic genomic and epigenomic alterations with prostate-specific TF hubs has not been performed. Using our novel computational approach, we first determined prostate-specific TF hubs and then analyzed WGS data from 188 primary PCa samples from the International Cancer Genome Consortium (ICGC) and other published studies [5, 9, 47] along with DNA methylation data from 333 samples from TCGA [3] to identify genetic and epigenetic alterations that alter hub expression and cause large-scale network changes, potentially driving the transformation of normal cells to a tumorigenic state. The large sample sizes used for this analysis provided the statistical power to detect and distinguish genes significantly altered by recurrent SNVs, SVs, or DNA methylation changes in their coding or non-coding regions from the background of random passengers.

Thus, we present a novel computational method that integrates genetic and epigenetic data from tumor samples and interprets the combined effects of coding and non-coding cis-regulatory regions significantly altered by SNVs, SVs, and DNA methylation on the tissue-specific regulatory network [5, 9, 48, 49].

## Results

**Fig. 1:**
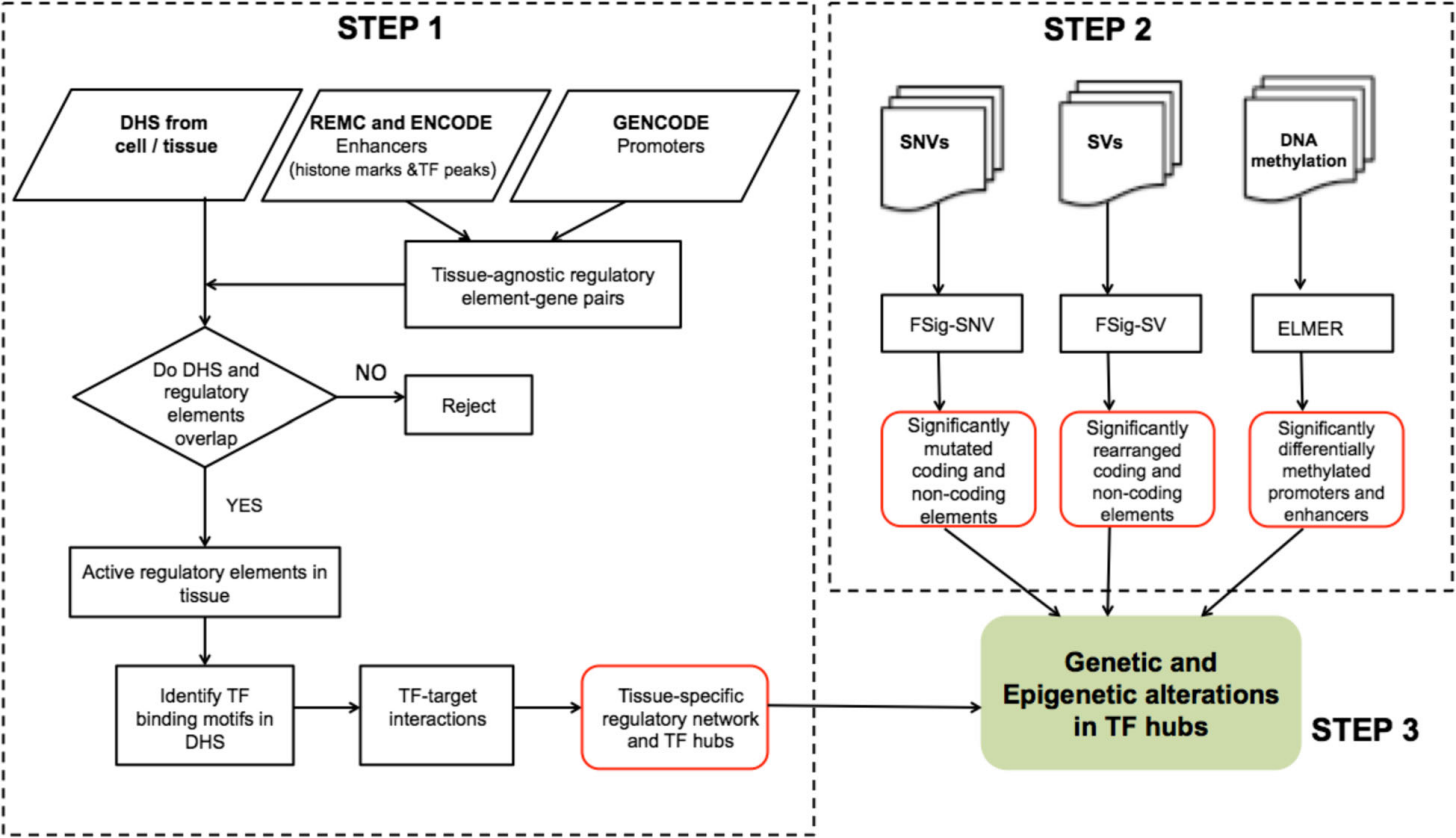
Flow chart of the three-step computational model for identifying genetic and epigenetic alterations causing large perturbations in the tissue-specific regulatory network. Step 1 is construction of the tissue-specific regulatory network using DHS data and identification of TF hubs. Step 2 identifies significantly mutated, rearranged, and differentially methylated coding and non-coding elements and step 3 combines the output of steps 1 and 2 and outputs the effect of genetic and epigenetic alterations on TF hubs. *DHS* DNase I hypersensitive sites, *REMC* Roadmap Epigenomics Mapping Consortium, *SNV* single nucleotide variant, *SV* structural variant, *TF* transcription factor

We developed a three-step computational model, RegNetDriver, for identifying genetic and epigenetic alterations causing large perturbations in tissue-specific regulatory network. The steps are: (1) construction of a tissue-specific regulatory network using DHS data and identification of TF hubs, (2) the identification of significantly mutated, rearranged, and differentially methylated coding and non-coding regulatory regions, and (3) the interpretation of the global impact of genetic and epigenetic alterations in the regulatory network (Fig. 1). We applied our computational model on genetic and epigenetic data from prostate tumor samples and identified regulatory drivers of prostate tumorigenesis.

### Construction of the tissue-specific regulatory network using DHS data and identification of TF hubs

#### Mapping TF–target gene regulatory interactions in prostate cells

Our pipeline for constructing the prostate network begins by identifying active cis-regulatory regions in prostate tissue. We identified 15,542 active promoters and 74,440 active enhancers using DHS in prostate epithelial cells (Methods). These numbers are consistent with current estimates of active promoters and enhancers in a tissue: ~10,000–15,000 promoters [50, 51] and ~44,000–294,000 enhancers [40, 52, 53] depending on the tissue type. To uncover TF-DNA binding sites in these active regulatory regions, we used the PIQ (Protein Interaction Quantification) tool [54] and a curated collection of sequence binding motifs for 617 TFs [55]. We find enrichment of 612 TF motifs in the active promoter and enhancer regions. This knowledge of enriched TF binding motifs was used to create TF–promoter and TF–enhancer edges in the regulatory network. The active prostate promoters and enhancers with enriched TF binding motifs were associated with their target genes to model promoter–target gene and enhancer–target gene interactions (Methods).

**Fig. 2:**
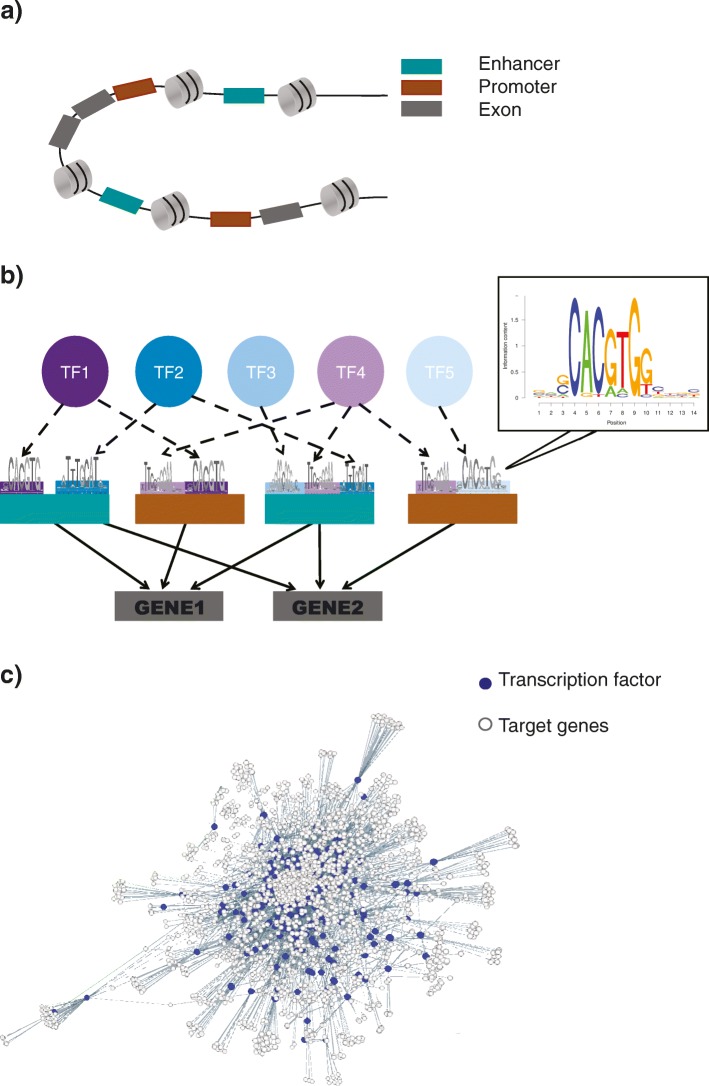
Schematic of DHS-based prostate regulatory network. **a** Identification of active promoters and enhancers: promoters and enhancers active in prostate tissue were identified by intersecting DHS from prostate epithelial cells with tissue-agnostic promoters and enhancers identified using functional annotations from ENCODE, REMC, and GENCODE. **b** Predict TF binding sites: binding sites of 617 TFs in active prostate promoters and enhancers were identified using 2065 motifs and PIQ. **c** Prostate Regulatory Network: the prostate regulatory network contains 17,087 genes (including 612 TFs), 15,542 promoters, 74,440 enhancers, and 1,209,599 unique directed TF–target gene interactions. For the purpose of presentation, we have limited the number of network edges. *DHS* DNase I hypersensitive sites, *PIQ* Protein Interaction Quantification, *REMC* Roadmap Epigenomics Mapping Consortium, *TF* transcription factor

One enhancer can regulate the expression of multiple genes [53] – we find in our network each enhancer is associated with an average of three genes (Additional file 1: Figure S1a). Similarly, multiple enhancers can regulate the expression of a gene. In our prostate regulatory network, each target gene is associated with an average of five active enhancers (Additional file 1: Figure S1b). Thus, using our pipeline, we generated an extensive prostate regulatory network (Additional file 2), which contains 17,087 genes (including 612 TFs) (Additional file 3: Table S1) and 1,209,599 unique directed TF–target gene interactions (Fig. 2).

#### Validation of prostate regulatory network

Although systematic validation of all interaction edges is difficult due to the large scale of the network and our limited understanding of the true network [56], a common approach is to use independent TF binding data from ChIP-Seq experiments [57]. We assessed the edges between TF and target genes using ChIP-Seq binding peaks for nine TFs (androgen receptor or AR [58], CTCF [59], ERG [29], ETS1 [60], ETV1 [61], GABPA [62], GATA2[63], NR3C1 [64], and TCF7L2 [65]). We report sensitivity, specificity, precision, and *F* score values for the predicted TF–target gene edges (Additional file 3: Table S2). For these nine TFs, we obtain an average sensitivity of 0.64 and specificity of 0.68. We note that ChIP-Seq data may not necessarily be the true gold standard due to the possibility of some edges labeled incorrectly as positive (non-specific TF binding) or negative (undetected regions), which can impact the sensitivity and specificity of our network [57].

**Fig. 3:**
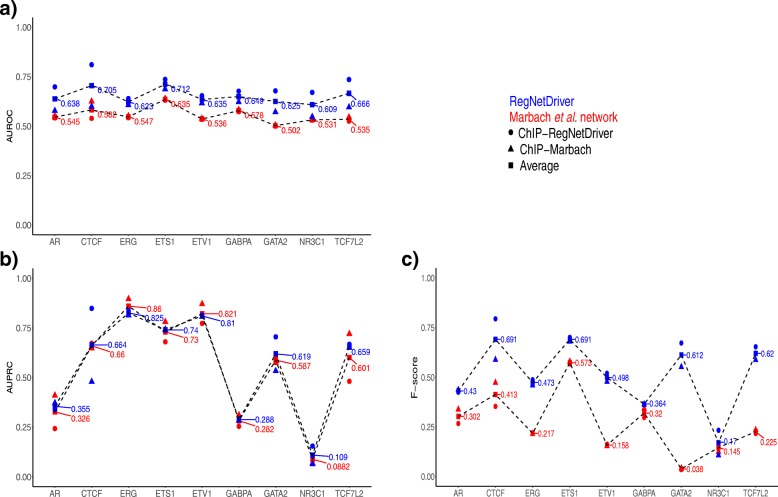
**a** Area under receiver operator characteristic curve, **b** Area under precision-recall curve (AUPRC), and **c** *F* scores for TF–target gene edges in the prostate epithelial networks of RegNetDriver (*blue*) and Marbach et al. (*red*). ChIP-Seq binding peaks of nine TFs (shown on the *x*-axis) and promoter–gene and enhancer–gene annotations from both networks are used for network evaluation. ChIP-Seq-based targets using RegNetDriver annotations are referred to as ChIP-RegNetDriver, shown in *circles* and targets using Marbach annotations are called ChIP-Marbach, shown in *triangles. Square* data points correspond to mean values computed using ChIP-RegNetDriver and ChIP-Marbach targets. *TF* transcription factor

We also compared the performance of our methodology to construct the tissue-specific regulatory network with another method [57]. Marbach et al. developed a network compendium comprising 394 cell type- and tissue-specific gene regulatory networks for human, including a prostate epithelial cell network using TF sequence motifs from ENCODE with promoter and enhancer activity data from the FANTOM project (http://regulatorycircuits.org) [57]. We compared the area under the receiver operator characteristic curve (AUROC), the area under the precision-recall curve (AUPRC), and *F* score values of the Marbach et al. predicted prostate TF–target genes with our network predictions (Fig. 3, Additional file 1: Figure S2 and Additional file 3: Tables S2 and S3). Overall, we find higher average AUROC, AUPRC and *F* scores from our network for the TF–target gene edges compared to Marbach et al. network (more details discussed in Methods). Also, we obtained overall higher AUROC and AUPRC values for RegNetDriver predicted TF–promoter and TF–enhancer edges in comparison to Marbach et al. (see Methods and Additional file 1: Figure S3).

The agreement of TF network edges with ChIP-Seq binding peaks and the better performance of our network in comparison to Marbach et al. provide validation for our prostate regulatory network predictions and demonstrate the potential of our DHS-based network construction algorithm to recapitulate global transcriptional regulatory interactions.

#### TF hubs are enriched for known cancer genes

We evaluated the degree centrality distributions of the genes in our regulatory network to identify the hubs (Additional file 1: Figure S4b, c). Hubs are defined as the highly connected TF genes (top 25% out-degree centrality) that regulate the expression of thousands of downstream genes [48]. Our prostate regulatory network consists of 153 TF hubs (Additional file 3: Table S4), which are significantly enriched for known cancer genes (odds ratio, OR = 2.24; *p* value = 0.00074) (Methods). In particular, TFs previously implicated in PCa are hubs in our network: ERG, ETV1, ETV4, NR3C1, NKX3-1, ETV3, NRF1, TP53, STAT3, ETV5, MYC, and ETV6 [3, 4, 6, 9, 66]. Other than known PCa genes, our list of TF hubs also contains novel candidates, which may have a role in prostate tumorigenesis (Additional file 3: Table S4).

Our prostate regulatory network represents the flow of information from TFs to target genes via regulatory elements and provides a list of regulatory TF hubs. Next, we investigated the genetic and epigenetic alterations in coding genes and non-coding cis-regulatory elements to identify the ones that are likely to impact TF-hub expression and cause large-scale network changes.

### Identification of significantly mutated, rearranged, and differentially methylated coding and non-coding regulatory regions

Different genetic and epigenetic events can trigger global remodeling of the prostate regulatory network. In an attempt to understand the combined effects of different events, such as SNVs, SVs, and DNA methylation changes on prostate transcriptional machinery, we analyzed data from primary prostate tumor samples to identify significantly mutated, rearranged, and differentially methylated regions, respectively.

#### Significantly mutated coding and non-coding regions (FSig-SNV method)

We performed a comprehensive analysis of somatic mutations in WGS data from 188 primary prostate tumor samples from ICGC and other published studies [5, 9, 47]. We developed the FSig-SNV (Functionally Significant Single Nucleotide Variants) method, which analyzes the somatic mutations in coding and non-coding (promoter and enhancer) regions to identify the elements that show more recurrent (present in multiple samples) and more functional mutations than expected randomly.

Our method combines the functional impact and positional recurrence of the variants to compute a composite score for each element. The functional impact score is computed using FunSeq2 [48, 67], a computational framework for annotating and calculating the functional impact score of coding and non-coding variants. FSig-SNV then compares the calculated composite score with a null background to compute *p* values. The null background is computed from random combinations of an equal number of SNVs in the element [68, 69] (Methods). The output of the method is a list of significantly mutated coding and non-coding elements that show a higher than expected frequency of functional mutations across multiple tumor samples.

**Fig. 4:**
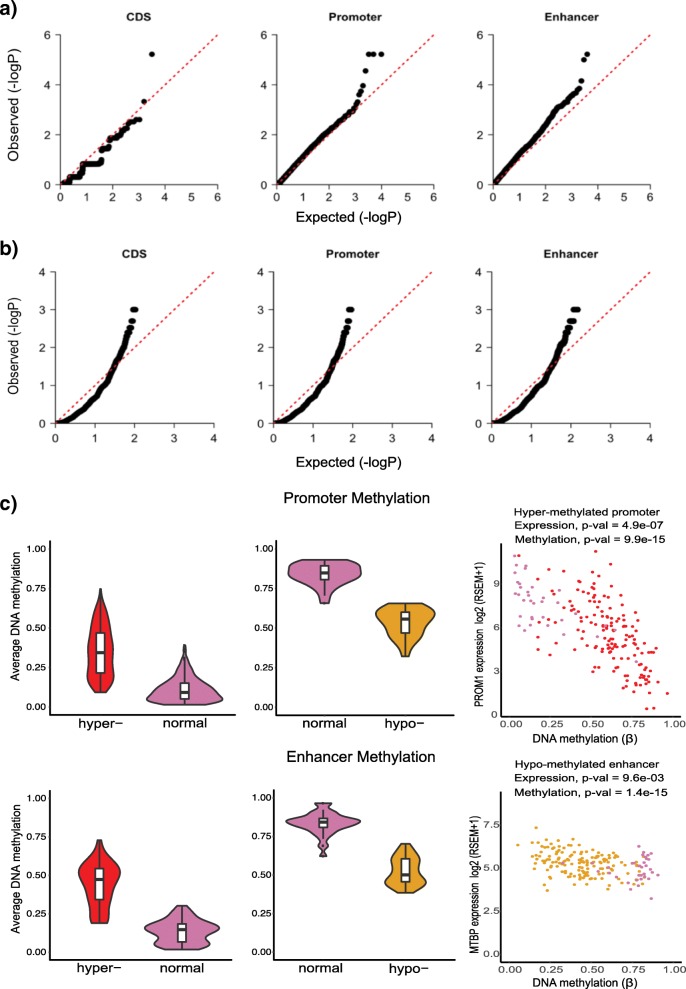
Significantly mutated, rearranged, and differentially methylated coding and non-coding regions. **a** QQ plots for significantly mutated coding sequence (CDS), promoters, and enhancers using FSig-SNV. **b** QQ plots for significantly rearranged CDS, promoters, and enhancers using FSig-SV. **c** DNA methylation changes and their association with gene expression. Left and middle: Significantly differentially methylated promoters and enhancers. Violin plots show a significant difference in methylation levels (Wilcoxon test *p* value < 0.05) at hyper- and hypo-methylated cis-regulatory regions (promoters and enhancers) between 333 tumor and 35 normal samples. Right: Scatter plots show examples of genes differentially expressed due to a hyper-methylated promoter (*PROM1*) and a hypo-methylated enhancer (*MTBP*) in tumor vs. normal samples. *MTBP* and *PROM1* have been reported to be differentially expressed in *TMPRSS2*:*ERG* positive prostate tumors [122]. A significant decrease in *PROM1* expression in 152 *TMPRSS2*:*ERG* fusion positive TCGA samples (*red*) vs. normal (*pink*) is associated with a hyper-methylated promoter (cg04203238). A significant increase in *MTBP* expression in *ERG* fusion positive samples (*yellow*) is associated with hypo-methylation in an enhancer (cg11476306). *CDS* coding sequence

QQ plots show that the *p* values calculated using the FSig-SNV method for coding regions, promoters, and enhancers follow the expected uniform distribution (Fig. 4a). Using FSig-SNV, we identified the coding region of a gene (*SPOP*), the promoters of three genes (*NBPF10*, *PDE4DIP* and *ZNF595*), and the enhancer of a gene (*HM13*) to be significantly mutated (Fig. 4a). Consistent with previous PCa studies [3, 5, 6], *SPOP* is nominated by FSig-SNV as the most significantly mutated coding candidate (8% in our dataset).

In total, 42% of the tumor samples harbor at least one mutation in a significantly mutated element: the coding region of *SPOP* or the promoter of *PDE4DIP*, *NBPF10*, or *ZNF595*, or the enhancer of *HM13*. We note that while our analysis reveals the non-coding regions of *PDE4DIP*, *ZNF595*, and *HM13* as significantly mutated in PCa for the first time, their coding regions have been previously implicated in prostate or other cancers [70–73].

#### Significantly rearranged coding and non-coding regions (FSig-SV method)

Given the important role of oncogenic fusions and chromosomal rearrangements in PCa, we next identified coding and non-coding regulatory regions significantly altered by SVs across multiple tumor samples. Briefly, our FSig-SV (Functionally Significant Structural Variants) method begins with the identification of elements affected by deletion, insertion, duplication, inversion, or translocation events. For each element, it counts the number of samples that exhibit an SV event and compares them with a null background generated by randomly shuffling SV breakpoints, keeping SV length and overall number of SVs in each chromosomal arm constant (Methods). The output of the method is a list of coding and non-coding elements that are rearranged in more samples than expected randomly. We analyzed somatic SVs, which include copy number variants (such as deletions and duplications), and copy number neutral variants (such as inversions, translocations, and other complex rearrangements), from 188 PCa whole-genomes from ICGC and other published studies [5, 9, 47] (as discussed under FSig-SNV). Figure 4b shows the QQ plots for coding regions, promoters, and enhancers.

We identified 168 genes with significantly rearranged coding regions. This includes genes already implicated in prostate tumorigenesis, such as *PTEN*, *ERG*, *TMPRSS2*, *TP53*, and *FOXP1*. In addition, we identified 169 genes with significantly altered promoters and 187 genes with significantly altered enhancers. Overall, 264 genes exhibit significant rearrangements in their coding or non-coding regions. Thus, using data from 188 whole genomes, we find that a much larger number of coding genes and non-coding regulatory elements are affected by SVs (524) than SNVs (5) in PCa (Table 1 and Additional file 4: Tables S5–S7).

**Table 1:**
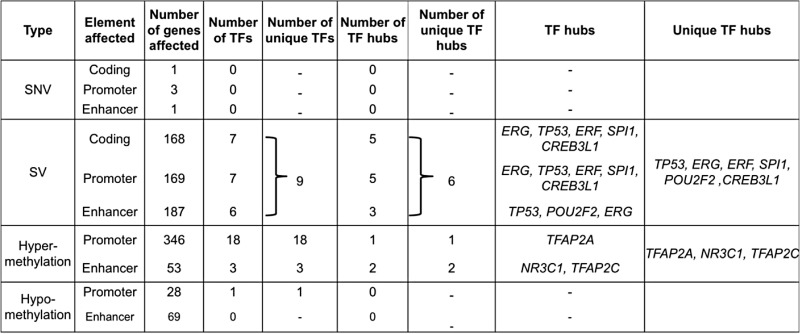
Genes affected by genetic and epigenetic alterations in their coding and non-coding elements

*SNV* single nucleotide variant, *SV* structural variant, *TF* transcription factor

#### Significantly differentially methylated promoters and enhancers

DNA hyper-methylation at cis-regulatory regions is mostly associated with down-regulation of gene expression, and hypo-methylation with up-regulation [74]. TCGA PCa study showed that genes silenced due to promoter hyper-methylation are significantly enriched for genes previously known to be differentially expressed in PCa [3]. Using HumanMethylation450 (HM450) array data corresponding to 333 TCGA primary prostate tumor samples and the ELMER (Enhancer Linking by Methylation/Expression Relationships) package [52], we identified 4,591 hyper- and 1,177 hypo-methylated promoter probes and 603 hyper- and 267 hypo-methylated enhancer probes (Fig. 4c). Using mRNA expression data from the tumor samples, we determined putative target genes whose expression is modulated by differential methylation of probes (Methods). We found 346 genes significantly associated with hyper-methylated promoters, 28 genes with hypo-methylated promoters, 53 genes linked with hyper-methylated enhancers, and 69 genes with hypo-methylated enhancers (Wilcoxon rank-sum test, *p* value < 0.01) (Additional file 4: Table S8). In total, 496 genes are associated with differentially methylated regulatory regions and are significantly enriched for genes found to be differentially expressed in PCa relative to normal samples (Methods, Fisher’s exact test, *p* value = 3.77 × 10^-9^, OR = 1.74). In Fig. 4c, we show examples of genes whose expression is associated with methylation at their regulatory regions. Thus, we find that an even larger number of genes are affected by differential methylation than by SVs or SNVs (Table 1).

The list of genes with significantly mutated or rearranged coding or regulatory regions and those with differentially methylated promoters or enhancers is provided in Additional file 4: Table S5. This list contains both known cancer genes and novel candidates that may play a role in prostate tumorigenesis. Next, we analyzed the impact of these significant genetic and epigenetic perturbations on the prostate regulatory network to identify the regulatory TF drivers.

### Interpretation of the global impact of genetic and epigenetic alterations in the regulatory network

The results discussed under “Identification of significantly mutated, rearranged, and differentially methylated coding and non-coding regulatory regions” show the impact of genetic and epigenetic alterations on individual genes. We further analyzed the impact of these alterations in the context of the prostate regulatory network.

#### Common effects of distinct genetic and epigenetic changes on gene expression

**Fig. 5:**
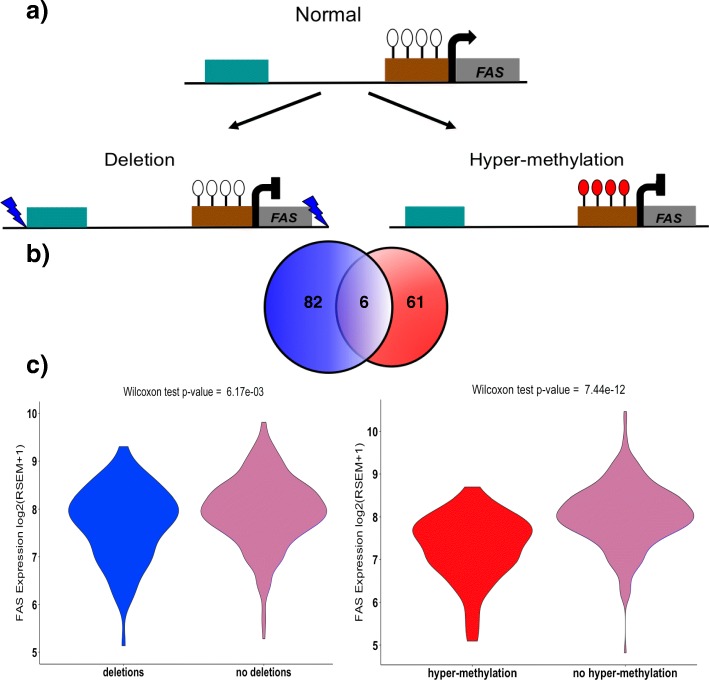
Effect of genetic and epigenetic alterations on gene expression. **a** Schematic showing the effect of deletion and hyper-methylation of the promoter region on expression of *FAS* (enhancers are shown in *bluish green* and the promoter in *brown*). **b** Venn diagram showing the number of samples affected by deletion (*blue*) and hyper-methylation of the promoter (*red*). **c** Violin plots showing the change in expression of *FAS* in tumor samples with deletion (*n* = 88) and tumor samples with hyper-methylated promoters (*n* = 67)

To understand whether dysregulated gene expression can be a consequence of the combined effects of genetic and epigenetic changes, we analyzed the list of genes significantly altered by SNVs, SVs, and differential methylation. Overall, 757 genes are significantly affected by genetic or epigenetic alterations. Out of these genes, only three genes are affected by both genetic and epigenetic events: *FAS*, *FAM3B*, and *TNFSF13*. These three genes are in deleted loci covering the entire gene body and exhibit hyper-methylation in their promoter regions (Fig. 5a). To analyze the combined effects of these different events on gene expression, we used RNA-Seq, DNA methylation, and deletion data from 333 TCGA tumor samples. For *FAS*, we find 88 samples have undergone gene deletion and 67 samples show significant hyper-methylation in the promoter region. However, 93% of these samples have the *FAS* gene altered by either methylation changes or deletion and only six samples exhibit both deletion of the gene and promoter hyper-methylation (see the Venn diagram in Fig. 5b). This high percentage of unique samples altered by each event suggests that genetic and epigenetic events independently lead to *FAS* dysregulation in PCa samples. Indeed, we find down-regulation of *FAS* in samples with deletions vs. without (*p* value = 0.0061) and in samples with promoter hyper-methylation vs. without (*p* value = 7.43 × 10^-12^) (Fig. 5c and Additional file 1: Figure S5). *FAS* plays a central role in programmed cell death and is important for regulating cell proliferation and tumor-cell growth [75, 76]. We note that germline polymorphisms leading to its dysregulated expression have been previously associated with a high risk of cancer, including PCa [75, 77, 78].

We obtained similar results for *FAM3B* and *TNFSF13*, i.e., a high percentage of unique samples altered by either promoter hyper-methylation or deletion (Additional file 1: Figure S6a and b). *FAM3B* and *TNFSF13* also have known implications in cancer [79, 80] and their altered expression may have some role in prostate tumorigenesis. These results demonstrate that distinct genetic and epigenetic events can independently lead to the same effects on gene expression in PCa, though it is not common.

#### SVs have a stronger influence on TF hubs than SNVs and DNA methylation

**Fig. 6:**
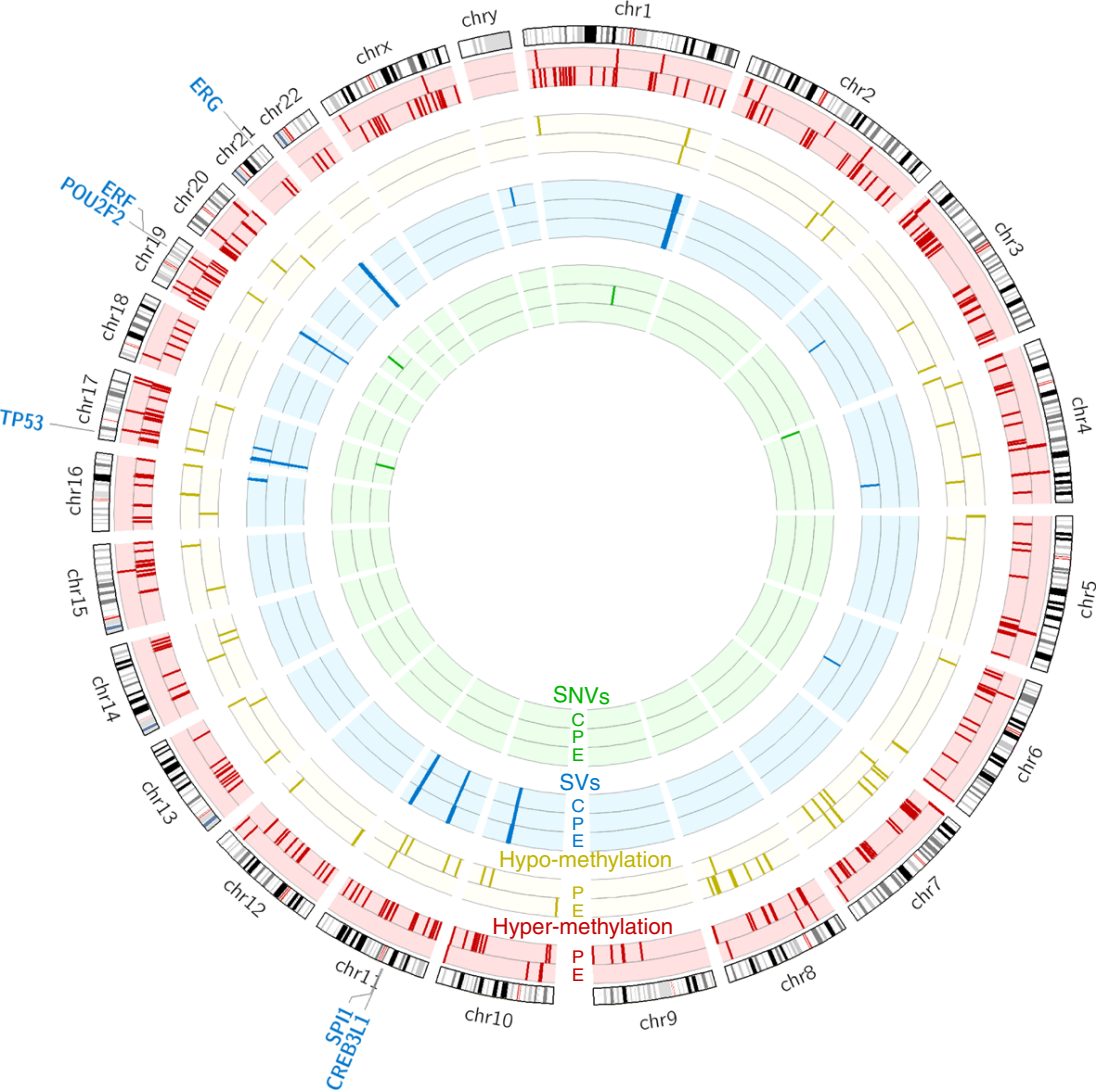
Genomic landscape of prostate cancer. *Green bars* represent genes with significant SNVs in the coding sequence (*C*), promoter (*P*), and enhancer (*E*) regions, *blue bars* correspond to genes with significant SVs in coding sequence, promoter, and enhancer regions, *yellow bars* show genes with hypo-methylated promoters and enhancers, and *red* indicates genes with hyper-methylated promoters and enhancers. The genomic location of six TF hubs (*blue*) affected by SVs is shown in the outer ring. *TF* transcription factor

Overall, we observe more TF genes are affected by methylation changes than SVs or SNVs (Table 1 and Fig. 6). Altogether, 22 TF genes show significant differential methylation in promoter or enhancer regions, while nine are significantly altered by SVs and none by SNVs (Table 1). Although observed for the first time in the genome-wide analysis of TF genes, this is consistent with previous PCa studies, which report a low mutation rate in prostate tumors compared to other tumor types and found a high recurrence of SVs and DNA methylation changes [3, 25, 81]. However, going one step further, we observe a stronger influence of SVs on TF hubs compared to methylation changes or SNVs. Out of nine TF genes altered by SVs, six are hubs (*ERG*, *TP53*, *ERF*, *SPI1*, *CREB3L1*, and *POU2F2*), whereas among 22 TFs with significant methylation changes, only three are TF hubs (*TFAP2A*, *TFAP2C*, and *NR3C1*). Thus, we find that TFs altered by SVs are significantly enriched for hubs compared to TFs altered by methylation changes (Fisher’s exact test: OR = 11.28, *p* value = 0.0068) (Table 1). We note that apart from *TP53* and *ERG*, which are known PCa genes, our list of TF hubs altered by genomic and epigenomic changes include *TFAP2A* [82], *CREB3L1* [83], and *ERF* [84], which have some reported implications in prostate tumorigenesis.

Dysregulation of the central nodes in the network is likely to produce global effects on prostate transcriptional machinery and these numbers show that a larger number of TF hubs are likely to be dysregulated by SVs compared to methylation changes. Indeed, we find that five out of six TF hub genes affected by recurrent SVs show differential expression between normal and tumor samples (*ERG*, *TP53*, *ERF*, *CREB3L1*, and *POU2F2*) (see Methods for *p* values, interaction edges shown in Additional file 1: Figure S7). Altered expression of *ERG* and *TP53* due to gene fusions and deletions has already been shown to play an important role in prostate tumorigenesis [85, 86]. We propose that the dysregulated expression of the remaining three TF hubs identified in our study (*ERF*, *CREB3L1*, and *POU2F2*) can also lead to large-scale changes in the prostate regulatory network, which in turn can play an important role in the transformation of normal cells to a tumorigenic state.

Next, we performed functional validation to understand the role of differential *ERF* expression in prostate tumorigenesis.

#### Functional validation of novel prostate regulatory driver, ERF

ERG and ERF are members of the ETS family of TFs. ERG is a transcriptional activator, whereas ERF is a transcriptional repressor whose interaction with ETS binding sites can suppress ETS-associated tumorigenesis [87, 88]. We observe a significant increase in *ERG* expression and a significant decrease in *ERF* expression due to SVs (Additional file 1: Figure S8). Also, we find significant enrichment of common binding targets for ERG and ERF in our prostate regulatory network (common targets = 3,061, Fisher’s exact test OR = 106.72 and *p* value < 2.2 × 10^-6^). Based on these results, we hypothesized that a decrease in *ERF* expression in prostate tumor samples can cause activation of the ETS transcriptional program similar to ERG activation. To test our hypothesis, we used the *ERF* gene expression signature generated from the lentiviral shRNA knockdown and RNA-Seq analysis of immortalized prostate epithelial cell line (LHS-AR) and a PCa cell line that harbors oncogenic *ERG* rearrangement (VCaP) [89]. Among the top 100 up-regulated genes due to *ERF* knockdown in the VCaP cell line (see Methods), 63 are present in our prostate regulatory network. Genes up-regulated due to *ERF* knockdown in the VCaP cell line are significantly enriched among both ERF and ERG network predicted binding targets [Fisher’s exact test OR = 2.38, *p* value = 0.00012 (ERF) and OR = 3.58, *p* value = 5.7 × 10^-7^ (ERG)]. Similar results were obtained for the LHS-AR cell line (Methods). The significant enrichment of the *ERF* gene expression signature in ERF and ERG binding targets validates our network predictions and supports our hypothesis that differential expression of *ERF* due to SVs can activate the ETS transcriptional program in prostate tumor samples.

This functional study demonstrates the strong potential of our computational method to identify novel regulatory drivers of tumorigenesis.

#### Network propagation of differential TF hub expression via methylation changes

Together, we find 7,675 differentially expressed genes in PCa samples relative to normal (Methods). However, it is unknown whether it is the genomic sequence changes or epigenetic alterations that initiate the cascade of gene expression changes in the prostate regulatory network.

The reversible nature of DNA methylation has attracted much attention towards understanding the mechanism by which it is regulated by TF binding. Whether methylation at TF binding sites is a consequence of TF gene expression or methylation gain is a cause for evicting TF remains unclear [90–92]. Based on the former hypothesis, multiple studies have proposed several models that have linked TF binding with acquisition or loss of methylation at regulatory elements [90–93]. We investigated the relationship between TF hub expression and DNA methylation at its binding sites. Using DNA methylation and expression data from 333 TCGA tumor samples, we calculated the correlation between the expression of six TF hub genes that are significantly altered by SVs (*ERG*, *TP53*, *POU2F2*, *SPI1*, *CREB3LI*, and *ERF*) and average DNA methylation (β values) at differentially methylated probes within specific TF motifs. We find a significant association (*p* value < 0.05) between expression and average DNA methylation for three out of six TF hubs (*ERG*, *POU2F2*, and *SPI1*). This significant correlation hints towards DNA methylation as a dynamic process that can be programmed to respond to changes in TF expression (Methods) (Additional file 1: Figure S9). Moreover, these results suggest that changes in TF hub expression due to SVs can trigger epigenetic changes at their binding sites in cis-regulatory regions, which would in turn lead to differential expression of their associated genes.

In our study, we observe that more TF hubs are perturbed by SVs than SNVs or DNA methylation, even though a larger number of genes are affected by epigenetic alterations. Significant associations between TF hub expression and methylation changes indicate a possible mechanism for network propagation of differential hub expression. We propose SVs as the initiators that perturb the expression of hubs and DNA methylation changes as the propagators of gene expression changes in the prostate regulatory network. This model would explain the larger number of TF hubs altered by SVs but overall a larger number of genes affected by methylation changes. However, since correlation does not imply direct causation, we functionally validated our hypothesis that altered expression of a TF hub can influence DNA methylation by Enhanced Reduced Representation Bisulphite Sequencing (ERRBS).

#### Functional validation of impact of SVs on global DNA methylation

**Fig. 7:**
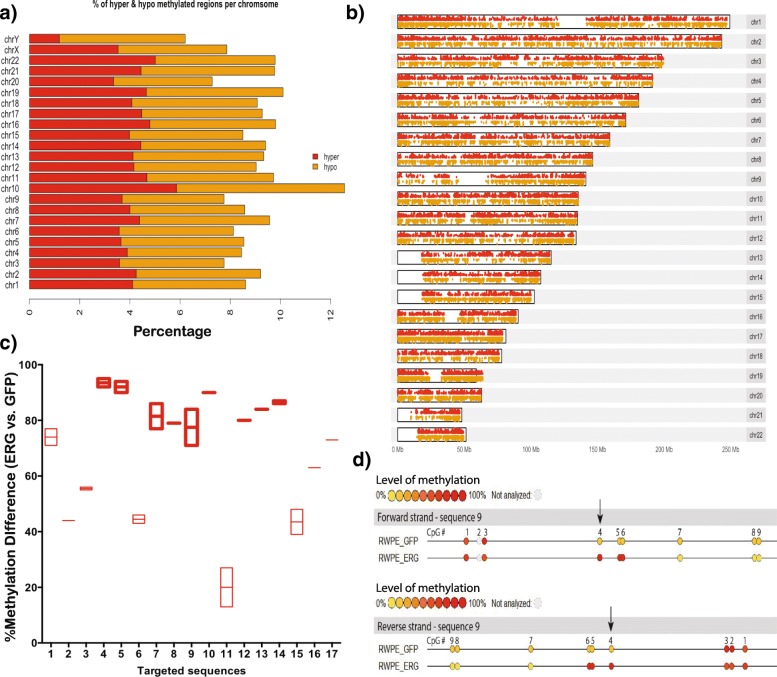
Overexpression of *ERG* causes global methylation changes. **a** Percentage of hyper-methylated (*red*) and hypo-methylated (*yellow*) regions out of all covered CpGs for each chromosome in the RWPE1-ERG cell line. **b** Chromosome ideogram showing sites of differential methylation in the RWPE1-ERG cell line. Hyper-methylated CpGs (*red*) and hypo-methylated CpGs (yellow). **c** Percentage methylation difference of 17 targeted sequences in the isogenic RWPE1 cells (+/- *ERG*), measured by the EpiTYPER MassARRAY assay. The *thick red outline boxes* represent the difference in methylation ratios between RWPE1-ERG and RWPE1-GFP measured on a sequence containing only the targeted CpG. The *red boxes with a light outline* correspond to an average of methylation ratios on a sequence containing two to four CpGs including the targeted one. For each targeted sequence, the methylation value has been measured in duplicate as shown in (**d**), except when the composition of the sequence did not allow it (single value for the sequences 2, 8, 10, 12, 13, 16, and 17). **d** Each CpG is represented by a *color-coded circle* depending on its methylation ratio. *Yellow* represents a low level of methylation, *red* represents a high level of methylation, and *grey* corresponds to unanalyzed CpGs. The values for the methylation ratio of CpG #4 in sequence 9 determined on both forward and reverse strands are shown in (**c**)

To substantiate our hypothesis that genetic changes such as SVs may be initiators that trigger changes in DNA methylation, we used the ERRBS assay for functional validation. ERRBS is a genome-wide single-base resolution DNA methylation assay that provides high coverage of CpGs [94–96]. Out of the six TF hub genes that are perturbed by SVs, *ERG* is rearranged at the highest frequency (~45% of tumor samples). *ERG* rearrangements result in its overexpression. To replicate the impact of SVs, we used two stable isogenic benign prostate epithelial cell lines (RWPE1), which only differ with respect to *ERG* expression [97]. We used RWPE1-GFP (control) and RWPE1-ERG cell lines to see if *ERG* overexpression can lead to global methylation changes. To estimate the methylation levels, we performed ERRBS for both RWPE1-ERG and RWPE1-GFP cells. ERRBS provided tenfold sequencing coverage on >2.5 million CpG sites genome-wide for each cell line. By analyzing methylation levels of all CpG sites, we observe an overall significant difference in the DNA methylation level of the RWPE1-ERG cell line in comparison to the control. There are 226,677 differentially methylated CpGs (*q* value < 0.01), including 105,720 CpGs that are hyper-methylated and 120,957 CpGs that are hypo-methylated in the RWPE1-ERG cell line vs. the control (Additional file 1: Figure S10). Figures 7a and b show the distribution of hyper- and hypo-methylated CpGs on each chromosome. Overall, we find ~9% of the genome is differentially methylated as a result of *ERG* overexpression in RWPE1-ERG cells. Furthermore, we used a site-specific methylation validation assay (EpiTYPER MassARRAY system) to specifically measure the methylation at the top 17 hyper-methylated CpGs identified from the ERRBS data in the RWPE1-ERG cells vs. GFP. We see a significant increase in the methylation for RWPE1-ERG cells with respect to GFP for all the 17 CpG sites, providing independent validation for the ERRBS results (Figs. 7c and d) (Wilcoxon test, *p* value = 3.28 × 10^-10^, Additional file 4: Table S9).

These results demonstrate that, within controlled isogenic conditions, global methylation changes are associated with *ERG* overexpression. Therefore, our findings provide further insights about the relation between TF hub expression and global methylation levels in prostate cells (discussed above), corroborating the role of genomic alterations in mediating changes in the epigenome.

### Computational pipeline to identify regulatory drivers

The protocol discussed in this work has been converted into a computational pipeline, RegNetDriver, to identify genetic and epigenetic alterations in the coding and non-coding regulatory regions of the tumor genomes and analyze their effects on tissue-specific regulatory networks (khuranalab.med.cornell.edu/RegNetDriver.html) (Fig. 1). In this study, we analyzed PCa genomes, but the pipeline can be easily used for different cancer types. We expect that analyses using the pipeline will reveal the varied roles of coding vs. non-coding and genetic vs. epigenetic alterations in diverse tumor types.

## Discussion

Previous studies have identified genetic and epigenetic differences in tumor vs. normal cells but translation of this information to an understanding of the processes involved in tumor development and progression still remains a major challenge. Progression of normal cells to a tumorigenic state involves differential expression of thousands of genes due to genetic and epigenetic changes [98, 99]. In this study, we present a computational framework to construct a tissue-specific regulatory network and use it to understand the global impact of genetic and epigenetic alterations on differential gene expression associated with cancer. To construct the prostate regulatory network, we utilized functional genomics data from ENCODE and REMC to associate prostate-specific promoters and enhancers to their target genes and predicted enrichment of TF motifs within these regulatory elements. Using this network, we identified TF hubs, which are predicted to regulate the expression of thousands of genes. Genetic and epigenetic changes that impact TF hubs can cause large-scale changes in the network, which in turn can drive the transformation of normal cells into a tumorigenic state [100, 101]. Identification and subsequent activation/inactivation of tumorigenic TF hubs by small molecules can impair tumor growth and development, as shown recently for ERG inhibition by dexamethasone [102]. Despite the challenges associated with considering TFs as drug targets [103], recent work by Gayvert et al. demonstrates the use of a computational drug-repositioning approach for targeting TF activity using small molecules [102]. Thus, TF hubs predicted to be associated with tumor progression by our approach can be potential novel therapeutic targets for cancer drug studies.

We identified 153 TF hubs in the prostate regulatory network. These are significantly enriched for known PCa genes and are predicted to play an essential role in maintaining the prostate transcriptional machinery. The prostate regulatory network and the associated hubs discussed in this work will be a useful resource for further investigations of other diseases involving the prostate tissue beyond the current study. In this study, we integrated our knowledge of the prostate regulatory network from normal cells with WGS and DNA methylation data from primary prostate tumors to analyze systematically how genetic and epigenetic alterations in coding and non-coding regions impact TF hubs and rewire the regulatory network in tumor cells. Using novel computational approaches (FSig-SNV and FSig-SV), we identify known PCa genes that are significantly mutated by SNVs (*SPOP*) or rearranged by SVs (*TMPRSS2*, *ERG*, *PTEN*, *CHD1*, *NKX3*-1, and *TP53*) and predict novel candidate genes with significant SNVs or SVs in their coding or non-coding regions (promoters and enhancers). Moreover, by analyzing DNA methylation profiles from tumor vs. normal samples, we identified genes with differentially methylated promoters and enhancers across multiple tumor samples. This repertoire of genes with substantial genetic and epigenetic alterations is a useful resource of potential drivers whose role in prostate tumorigenesis can be further explored. In our work, we focused on TF hubs that show enrichment of genetic or epigenetic changes in their coding or non-coding regions and can act as regulatory drivers.

We find that overall a higher number of genes exhibit significant differential methylation at promoters and enhancers than those that are significantly mutated or rearranged. However, TFs that are altered by SVs are significantly enriched for hubs compared to TFs altered by methylation changes, suggesting that SVs play a pivotal role in PCa development by causing larger perturbations in the regulatory network compared to SNVs and methylation changes. The TF hubs that show differential expression due to SVs are *ERG*, *TP53*, *POU2F2*, *CREB3LI*, and *ERF*. Out of these, *ERG* and *TP53* are known PCa genes and we propose that *POU2F2*, *CREB3L1*, and *ERF* can also play an important role in prostate tumorigenesis. Functional validation supports the hypothesis that *ERF* down-regulation leads to activation of the ETS transcriptional program that mediates cell invasion and tumor development. Very recently, a study of the exomes of African-American PCa patients suggested the role of *ERF* as a prostate tumor suppressor gene [89]. This study by Huang et al. provides independent validation of the predictions of our method and shows its immense utility in identifying novel cancer drivers.

Based on the DNA methylation changes observed on *ERG* overexpression, we propose a model to explain the consequences of TF hub dysregulation on the prostate regulatory network. In this model, crosstalk between TF hub expression (modulated by SVs) and DNA methylation allows global expression changes in the network. Previous studies have discussed multiple models to explain the crosstalk mechanism between TF expression and DNA methylation at its binding sites. One such model proposed that CpG islands at promoters can be protected from DNA methylation by TF binding [91]. In this model, higher TF expression would lead to increased recruitment of histone H3 lysine 4 (H3K4) methyltransferase, which protects the bound regions from methylation [91]. On the other hand, higher TF expression has also been associated with hyper-methylation [104, 105]. In this case, TF association with DNA methyltransferases promotes methylation at the bound regions. Thus, altered TF expression can be associated with both hypo- and hyper-methylation depending on the TF and the physiological context [91]. We propose that SVs may be the initiators that perturb the expression of hubs and DNA methylation changes can be the propagators of gene expression changes in the regulatory network. We note that the functional validation in this study analyzed the consequences of *ERG* and *ERF* expression changes on DNA methylation and downstream gene expression respectively, but future work can probe the direct impact of SVs on methylation and target gene expression.

The framework proposed in this study can be used to analyze other cancer types. We expect that our computational approach, RegNetDriver (provided at khuranalab.med.cornell.edu/RegNetDriver.html), will be extremely useful for analyzing the ~2,800 tumor whole genomes, transcriptomes, and epigenomes of 40 tumor types from the upcoming Pan-Cancer Analysis of Whole Genomes project [106].

## Conclusions

We provide a computational framework, RegNetDriver, to infer the global impact of tumorigenic genetic and epigenetic alterations in the tissue-specific network and identify regulatory cancer drivers. The application of our method on PCa data shows that SVs have a stronger regulatory impact than SNVs and methylation changes. TF hub expression modulated by SVs can, in turn, lead to methylation changes. We identify known regulatory drivers (*ERG* and *TP53*) and nominate novel TF genes (*ERF*, *CREB3L1*, and *POU2F2*) that are significantly rearranged across multiple PCa samples and predicted to cause dysregulation of thousands of genes. Functional validation of *ERF* supports its role in prostate tumorigenesis by activation of the ETS transcriptional program. RegNetDriver can be used to analyze other cancer types, and we expect SNVs, SVs, and methylation changes can play roles of varied importance in different tumor types and tissues.

## Methods

### The DHS-based prostate regulatory network

The gene expression program of a cell depends on the complex binding patterns of the TFs at the regulatory DNA [38, 54]. In our work, we have used DHS data from ENCODE for epithelial cells from the prostate (https://www.encodeproject.org/experiments/ENCSR000EPU/) along with GENCODE annotations [107], functional annotations from ENCODE [41], data from REMC [40], and position weight matrices for TF motifs from ENCODE [55] to construct a prostate regulatory network. In the following section, we describe the different steps involved in construction of the prostate network.

#### Identification of promoters and enhancers

We used tissue-agnostic regulatory elements and their association with target genes as described in the FunSeq2 protocol [67]. GENCODE v16 annotations were used and promoters are defined as being -2.5 kb from a transcription start site (TSS). To define tissue-agnostic enhancers, we used functional elements identified by ENCODE, which include regions of open chromatin associated with histone marks (H3K4me1, H3K4me2, and H3K27ac) and significantly enriched for ChIP-Seq identified TF-motifs [41]. Our list of tissue-agnostic enhancers also includes regions of open and accessible chromatin where transcription-related factors can easily bind even without cognate sequence motifs, which are defined as regions with a high occupancy of TFs (HOT) in the work of Yip et al. [108]. All regulatory elements that are at least 1 kb from the closest gene according to GENCODE annotations were annotated as enhancers [67]. To associate enhancers with their potential regulatory targets, we considered all candidate target genes within 1 Mb from enhancer regions. Correlations were calculated between the activity/inactivity signal at the enhancer region and expression at candidate target genes. Enhancer histone marks, such as H3K4me1 and H3K27ac, were considered as activity signals and DNA methylation data were used as inactivity signals. If there were significant correlation values, the matches were called enhancer–target gene pairs. Bisulfite sequencing, ChIP-Seq, and RNA-Seq data from REMC across multiple tissues were used to calculate correlations between activity/inactivity signals and expression data [67] (see Fu et al. [67] for more details).

To define active prostate promoters and enhancers, we intersected tissue-agnostic promoters and enhancers with DHS of prostate epithelial cells obtained from ENCODE (https://www.encodeproject.org/experiments/ENCSR000EPU/). We found 15,542 active prostate promoter regions and 74,440 enhancer regions. In the next step, we identified TF motifs enriched in these active prostate promoters and enhancers.

#### Prediction of TF binding motifs

We used the PIQ software [54] to predict TF binding in the DHS data. PIQ uses machine learning to normalize input DHS data and predict TF binding by detecting the shape and magnitude of DNase profiles specific to a TF [54, 109]. It takes as input DNase-seq experiment data, the genome sequence of the organism assayed, and a list of TF motifs represented as position weight matrices. We provided PIQ with DHS data from epithelial cells of the prostate, the human genome sequence, and 2,065 TF motifs for 617 TFs from ENCODE [55]. The output of PIQ is the probability of a TF binding at a motif in the genome. We selected all the active prostate promoters and enhancers with the predicted probability for the TF binding at the motif being ≥0.7. We found significant enrichment of 612 TF motifs in the active promoter and enhancer regions. This information was used to generate TF–promoter and TF–enhancer edges in our network. There are 629,263 TF–promoter edges and 267,415 TF–enhancer edges in our network. In the last step, we combined the results of steps 1a (Identification of promoters and enhancers) and 1b (Prediction of TF binding motifs) to put together a prostate regulatory network.

#### TF–target gene interactions

All the active prostate promoters and enhancers enriched for TF binding motifs are used to make connections between TFs and target genes. We used information of promoter–target gene and enhancer–target gene pairs from step 1a (Identification of promoters and enhancers) and TF binding motifs enriched in these regulatory elements from step 1b (Prediction of TF binding motifs) to make TF–target gene edges. The output of this three-step protocol is an extensive prostate regulatory network, where nodes represent TFs, target genes, and regulatory regions (promoters and enhancers). The edges correspond to interactions between these nodes, i.e., TF–promoter, TF–enhancer, promoter–target gene, and enhancer–target gene. The complete prostate network contains 629,263 TF–promoter edges, 267,415 TF–enhancer edges, 15,465 promoter–target gene edges, and 69,422 enhancer–target gene edges. We used a simplified form of the prostate regulatory network for the degree centrality analysis discussed in this work. The simplified prostate network contains only TF–target gene interactions. There are 17,087 genes in the prostate network, which includes 612 TFs. These 612 TFs are connected to target genes via 1,209,599 unique directed TF–target gene interactions.

### Validation of TF–target gene edges in the network

To validate the TF–target edges in the prostate regulatory network, we used ChIP-Seq data for nine TFs available from prostate tissues or cell lines (Additional file 3: Table S2). The dataset includes ChIP-Seq binding peaks for the AR from normal prostate tissue and CTCF binding peaks from prostate epithelial cells, while for the remaining TFs we used the available ChIP-Seq peaks from different PCa cell lines. The ChIP-Seq assay provides TF-bound regions in the genome. We intersected these TF-bound regions with all promoters and enhancers and connected them to the target genes using the same approach as defined in step 1a (Identification of promoters and enhancers). The ChIP-Seq-based targets are used as the gold standard and TF–target gene edges are defined as positive if they occur in ChIP-Seq-based targets and negative otherwise. Thus, in our analysis, true positives (TP) refers to predicted TF–target genes, which are also TF ChIP-Seq-based targets, false positives (FP) are predicted targets that are not ChIP-Seq-based targets and false negatives (FN) are ChIP-Seq-based targets not predicted by our network. Sensitivity (true positive rate or recall) = TP/(TP + FN), specificity (true negative rate) = TN/(TN + FP), precision (positive predictive value) = TP/(TP + FP), and *F* score = 2 × ((precision × recall)/(precision + recall)).

Our ChIP-Seq dataset includes AR and ERG binding peaks. AR and ERG play pivotal roles in prostate tumorigenesis [29, 85, 110]. AR activity is central for overexpression of most *ETS* genes such as *ERG* and *ETV1*, which are fused with androgen-regulated promoters in prostate tumors. Overexpression of *ERG* due to *TMPRSS2*:*ERG* fusion results in increased cell invasion and promotes PCa [29]. Differential AR activity has been shown to be associated with prostate tumor subtypes, such as those based on *SPOP* and *FOXA1* mutations [3]. Given the importance of AR in prostate, we validated our network-predicted AR edges using ChIP-Seq data. We overlapped 8,151 AR ChIP-Seq binding peaks from normal prostate tissue (GSM1358399) with 81,626 promoters for different gene isoforms and 769,538 enhancers with gene links. There are 3,759 ChIP-Seq-based AR targets. AR shares transcriptional targets with other members of the nuclear hormone receptor family [111]. Therefore, to compare AR binding targets predicted with RegNetDriver with AR ChIP-Seq-based targets, we considered both AR and NR3C1 motifs. We compared 8,931 AR targets in the prostate network against 3,759 AR ChIP-Seq-based targets from normal prostate tissue and obtained a sensitivity of 0.716, specificity of 0.618, and precision of 0.301 (Additional file 3: Table S2). Also, the predicted AR targets include some of the known target genes such as *FGF8*, *CDK1*, *CDK2*, *PMEPA1*, *TMPRSS2*, *SLC43A1*, *KLK3*, *KLK4*, *SLC45A3*, *CHD1*, *KIF1A*, *PRKCD*, *FZD9*, *CLDN4*, *MAFG*, *KIAA1217*, *OAT*, *TRPV3*, *SIRT7*, *GSTT2*, *HERC3*, *ELL2*, *CENPN*, *MED28*, *ACSL3*, *GNMT*, *ABCC4*, *PTGER4*, and *CRELD2* [3, 29, 85, 112, 113]. Moreover, it has been reported that AR and ERG co-occupy target loci in PCa cell lines and tissues [29]. We compared 8,931 AR edges with 5,103 ERG edges in the prostate network and found that 3,443 target genes are common. We found significant enrichment for common AR and ERG targets (hypergeometric distribution test, *p* value = 1.5 × 10^-151^). Additional file 3: Table S2 contains the results of network validation.

### Network comparison

We compared the performance of our prostate regulatory network with the prostate epithelial cell network of Marbach et al. for the nine TFs with available ChIP-Seq binding peaks. To evaluate TF–target gene edges, we overlapped the TF ChIP-Seq peaks with all promoters and enhancers and then evaluated them using the connections between promoter/enhancer and target genes obtained both from RegNetDriver (ChIP-RegNetDriver targets) and Marbach et al. annotations (ChIP-Marbach targets). Due to the unavailability of a true gold standard dataset for promoter–gene and enhancer–gene links, we evaluated TF–target edges for the nine TFs in RegNetDriver and the Marbach et al. network using both ChIP-RegNetDriver and ChIP-Marbach as gold standards. Figures 3a and b show the AUROC and AUPRC for TF–target genes edges in the prostate regulatory networks from RegNetDriver (blue) and Marbach et al. (red). In Fig. 3, circular data points represent network TF–target gene edges evaluated using ChIP-RegNetDriver targets and triangular data points represent network TF–target gene edges evaluated using ChIP-Marbach targets. We find higher AUROC and *F* scores for RegNetDriver for eight out of nine TFs using both ChIP-RegNetDriver targets and ChIP-Marbach targets (Figs. 3a, c). The mean values of AUROC and *F* scores computed using ChIP-RegNetDriver and ChIP-Marbach targets are higher for all nine TFs for RegNetDriver compared to Marbach (Fig. 3; blue square boxes represent mean values for RegNetDriver and red square boxes for Marbach). For AUPRC, we find that network annotations for generating ChIP-Seq-based TF–targets provide an additional advantage to the networks, which show higher AUPRC values for their respective annotation set. However, RegNetDriver shows higher mean AUPRC for seven out of nine TFs though the mean AUPRC values for RegNetDriver and Marbach are more similar than AUROC or *F* scores (Fig. 3b). Overall, these results demonstrate the ability of a DHS-based network to recover more reliably true prostate regulatory interactions.

We also evaluated the predicted TF–promoter and TF–enhancer edges using ChIP-Seq binding peaks. We overlapped ChIP-Seq peaks for the nine TFs with all promoters and enhancers. We defined an edge as positive if there was a TF ChIP-Seq peak overlapping the regulatory element and negative otherwise. Additional file 1: Figure S3 shows the AUROC and AUPRC for TF–promoter and TF–enhancer edges in the prostate networks of RegNetDriver and Marbach. For eight out of nine TFs, we see higher AUROC and AUPRC for RegNetDriver TF–promoter edges in comparison to the Marbach et al. network. The AUROC is also higher for eight TFs for TF–enhancer edges in RegNetDriver while AUPRC is higher for five TFs and very similar (maximum difference = 0.05) for four TFs (Additional file 1: Figure S3). We provide details of the files used for evaluation of the Marbach et al. prostate regulatory network in the Additional file 1: Supplementary text.

### Regulatory TF hubs are enriched for known cancer genes

We used the prostate regulatory network to identify TF hubs, which are defined as the top 25% of the highest out-degree TFs [48]. Out-degree is the number of outgoing edges per node. There are 153 TF hubs in our prostate regulatory network (Additional file 3: Table S4). Next, we looked for cancer genes in our list of TF hubs. In total, 573 genes were annotated by the Sanger Center as causally implicated in oncogenesis (Cancer Gene Census; http://cancer.sanger.ac.uk/census/) and 31 out of 153 TF hubs are known cancer genes. Fisher’s exact test was applied to test the hypothesis that TF hubs are significantly enriched for known cancer genes (OR = 2.24; *p* value = 0.00074).

### Significant genomic alterations

To identify significantly mutated coding and non-coding elements (promoters and enhancers), we developed a novel computational method called FSig-SNV, which uses the functional impact and positional recurrence of the variants present in both coding and non-coding regions. For functional annotation, the method uses FunSeq2 [67] to annotate and calculate the functional bias score of each variant. FunSeq2 uses a weighted scoring scheme that takes into account features such as the functional annotation of variants; the presence of variants in sensitive, ultra-sensitive, ultra-conserved, and HOT regions; the nucleotide-level impact of regulatory variants, which includes the motif-breaking and motif-gaining score; and the network properties of variant-associated genes [48, 67]. This weighting method provides FunSeq2 with the ability to prioritize cancer somatic functional mutations better relative to other methods [67, 114].

The positional recurrence of a variant is defined as the number of tumor samples with the same mutated position. At each mutated position (*i*) in the genome, we multiplied the positional recurrence (*W*) and functional impact score (*FS*) to get a positional FSig-SNV_pos_ score:

$$ \mathrm{FSig}\hbox{-} {\mathrm{SNV}}_{\mathrm{pos}} = {W}_i \times F{S}_i. $$

A summation of FSig-SNV_pos_ for all the variants in an element (coding, promoter, or enhancer) is defined as the FSig-SNV score:

$$ \mathrm{FSig}\hbox{-} \mathrm{S}\mathrm{N}\mathrm{V}\kern0.2em \mathrm{score}\kern0.5em =\kern0.5em {\displaystyle {\sum}_{i=0}^n}{W}_i\times F{S}_i $$

where *n* is the total number of variants in an element.

To assess the significance of the FSig-SNV score for each coding and non-coding element, we perform a permutation test. In the permutation test, the observed FSig-SNV score for an element is compared with a null distribution of permuted FSig-SNV scores and the *p* value is calculated. Null distributions specific to coding and non-coding elements are generated by randomly sampling the same number of positional scores (FSig-SNV_rpos_ scores, where rpos refers to random positional scores) within the same genomic element. Next, we sum FSig-SNV_rpos_ scores into an aggregate FSig-SNV_random_ score. Studies have shown that the mutation rate varies across the mammalian genome and late-replicating regions are associated with a higher mutation rate [68, 115, 116]. The *p* value of the test element is calculated using

$$ P=\frac{1+{\displaystyle {\sum}_{n=1}^N}F\left({X}_n\ge {X}_0\right)}{N+1}. $$

The output of function *F* is 1 when *X*_*n*_ is bigger than or equal to *X*_*0*_, otherwise it is 0. *N* is the number of sampling iterations (default = 10^6^). *X*_*0*_ is the observed FSig-SNV score for a coding or non-coding element of a gene and *X*_*n*_ is the FSig-SNV_random_ score. We use the Benjamini and Hochberg method for multiple hypothesis testing (*q* value ≤ 0.05). The output of the method is a list of genes with significantly mutated coding, and promoter or enhancer elements.

We developed the FSig-SV method to identify significantly rearranged coding and non-coding elements. The term “rearranged” refers to SVs, including deletions, insertions, duplications, inversions, and translocations. To find rearranged regions within each chromosome, we first generated a list of coding and non-coding regions (promoter and enhancer) altered by different SVs. Next, we counted the number of samples with SVs affecting the listed coding and non-coding regions. In this way, we know the number of samples with rearranged coding and non-coding regions in each chromosome. To identify significantly rearranged regions in each chromosome, we simulate a background distribution of SVs by randomly shuffling breakpoints keeping the total number of samples, number of SVs per chromosome, and length of SVs constant. For each coding and non-coding element within a chromosomal arm, FSig-SV compares the number of samples affected in simulated data with that observed and computes *p* values. The formula for the *p* value calculation is the same as that discussed above for FSig-SNV, where *N* corresponds to the number of sampling iterations (default 10^3^), *X*_0_ is the number of SVs in a coding or non-coding element, and *X*_*n*_ is the number of random SVs in the element. The Benjamini and Hochberg method was used for multiple hypothesis testing (*q* value ≤ 0.01). The output of the method is a list of genes with significantly rearranged coding or non-coding elements.

For the above analysis, we used somatic SNVs and somatic SVs from WGS data of 188 primary prostate adenocarcinomas. These 188 primary PCa samples include 124 PRAD-CA samples from ICGC [47] (https://dcc.icgc.org/projects/PRAD-CA), 57 samples from the work of Baca et al. [9], and seven samples from Berger et al. [5]. We used 912,004 SNVs and 3,888 SVs from PRAD-CA samples (*n* = 124) and 350,049 SNVs and 6,465 SVs (*n* = 64) from the Baca et al. and Berger et al. samples.

### DNA methylation

We used TCGA level 3, Illumina Infinium HumanMethylation450 (HM450) array data corresponding to 333 primary prostate samples, 35 normal samples, and the ELMER package [52] for identifying differentially methylated promoter and enhancer regions. To define promoter probes, we used the promoter definition of being -2.5 kb from a TSS (as discussed above in Identification of promoters and enhancers section). Out of 485,512 array probes, 167,284 were defined as promoter probes and 20,094 probes overlapped with enhancer regions (see Identification of promoters and enhancers section for the enhancer definition). The amount of DNA methylation at each CpG is referred to as the β value, where β = *M*/(*M* + *U*) and *M* is the methylated allele intensity and *U* is the unmethylated allele intensity. The ELMER package uses a *t*-test to identify promoter and enhancer probes that are significantly hyper-methylated or hypo-methylated relative to normal samples (*n* = 35 samples). To identify hypo-methylated probes, ELMER compares the 20% of normal samples with the lowest methylation to the 20% of tumor samples with the lowest methylation and performs an unpaired one-tailed *t*-test. Similarly, to identify hyper-methylated probes, it compares the 20% of the highly methylated normal and tumor samples [52]. For additional stringency, ELMER considers a probe as differentially methylated if the methylation difference is greater than 0.3 (|μ_normal –_ μ_tumor_| > 0.3) and the one-tailed *t*-test *q* value < 0.01. Apart from identifying differentially methylated promoter and enhancers, ELMER correlates the state of these regions with the expression of the nearby genes to identify transcriptional targets [52]. The output of the package is a list of significantly differentially methylated promoter and enhancer probes (*q* value ≤ 0.01) and their significantly associated target genes (*q* value ≤ 0.01).

To generate scatter plots for visualizing the effect of the expression of TF hubs on global methylation, we used level 3 HM450 PRAD DNA methylation data for 333 TCGA prostate samples and log2-transformed level 3 PRAD TCGA RNA-Seq RSEM data.

### Common effects of genetic and epigenetic alterations on differential gene expression

To analyze that impact of SVs and promoter DNA hyper-methylation on the expression of *FAS*, *FAM3B*, and *TNFSF13*, we first identified TCGA tumor samples with these alterations. Out of 333 tumor samples, 88 samples had *FAS* deleted, 92 samples had *FAM3B* deleted, and 113 had *TNFSF13* deletions. To identify hyper-methylated samples for each gene, we selected the top 20% of the tumor samples, i.e., 67 tumor samples with the highest methylation at promoter probes cg26478401 for *FAS*, cg22612764 for *FAM3B*, and cg13829089 for *TNFSF13*. To be consistent with the ELMER package, we used a 20% cutoff, which is used to identify differentially methylated promoter and enhancer probes with respect to normal. The 20% cutoff allows identification of molecular subtypes making up minority cases, while providing enough statistical power for predictions [52]. To generate the Venn diagram (Fig. 5b), we segregated tumor samples into three categories: samples with gene deletion, hyper-methylation in promoter regions, and samples with both deletion and hyper-methylation. To analyze the effect of deletion and differential methylation on expression, we used level 3 PRAD TCGA RNA-Seq data corresponding to 333 tumor samples. The Wilcoxon rank sum test was used to compare the distribution of samples with and without deletions/hyper-methylation.

### Differential gene expression

Level 3 PRAD TCGA RNA-Seq data for 333 tumor and the adjacent 27 normal samples were used for finding differentially expressed genes between normal and tumor. We performed the Wilcoxon rank sum test to identify differentially expressed genes between normal and tumor samples at a false discovery rate threshold of 0.0001. We obtained 7,675 genes that are either up- or down-regulated with respect to normal.

### SVs have stronger influence on TF hubs

We applied Fisher’s exact test to check whether TFs altered by SVs are significantly enriched for TF hubs compared to TFs altered by methylation changes. Out of 31 TFs significantly altered by genetic and epigenetic changes, 22 TFs have significant differential methylation in promoter or enhancer regions, nine are significantly altered by SVs in coding or non-coding region, and none by SNVs. Among 22 TFs, three are hubs, while six are hubs among the nine TFs affected by SVs. We found that TFs altered by SVs are significantly enriched for hubs compared to TFs altered by methylation changes. Also, five out of the six hub genes affected by SVs are differentially expressed between tumor and normal samples (*ERG p* value = 0.02, *TP53 p* value = 8.61 × 10^-6^, *ERF p* value = 0.05, *CREB3L1 p* value = 2.5 × 10^-11^, and *POU2F2 p* value = 4.02 × 10^-10^).

Copy number variants in PCa have been shown to be associated with disease recurrence and metastasis [3, 81, 117]. We stratified 188 WGS and 333 TCGA tumor samples with deletions in the five TF hubs by Gleason score and observed enrichment for Gleason score 8 or higher in TCGA samples (OR = 2.13, *p* value = 0.003). The absence of this enrichment in WGS data could be due to depletion of tumors with a high Gleason score in ICGC samples (Additional file 4: Table S10).

### ERF signature

To generate the *ERF* signatures, we analyzed the RPKM RNA-Seq profiles of *ERF* shRNA knockdown in VCaP cell lines [89]. Briefly, we independently rank genes according to the difference of means between the shERF-infected VCaP and control samples. The same procedure was performed for the LHS-AR cell line. The top 100 up-regulated genes in LHS-AR and VCaP cell lines are provided as part of the supplementary information (Additional file 4: Table S11). We performed Fisher’s exact test to assess whether *ERG* and *ERF* binding targets in the prostate regulatory network are enriched for genes up-regulated due to ERF knockdown in VCaP and LHS-AR cell lines. Out of 17,087 prostate network genes, 5,103 are *ERG* binding targets and 3,327 are *ERF* target genes. Among the top 100 up-regulated genes due to *ERF* knockdown in VCaP, 63 are present in our prostate regulatory network, 38 are *ERG* binding targets, and 25 are *ERF* targets. Similarly, among the top 100 up-regulated genes in LHS-AR, 76 are present in the prostate network, 36 are *ERG* target genes, and 21 are *ERF* targets [VCaP: Fisher’s exact test OR = 2.38, *p* value = 0.00012 (*ERF*) and OR = 3.58, *p* value = 5.7 × 10^-7^ (*ERG*); LHS-AR: Fisher’s exact test OR = 1.64, *p* value = 0.05 (*ERF*) and OR = 2.12, *p* value = 0.0014 (*ERG*)]. We would like to note that a change in *ERF* expression impacts the downstream transcriptional program through activation of both direct and indirect binding targets. As it is hard to compute indirect binding targets from our network, we restrict our analysis to direct targets only.

### TF expression and DNA methylation

We calculated the Spearman correlation between TF hub expression (*ERG*, *TP53*, *POU2F2*, *SPI1*, *CREB3LI*, and *ERF*) and DNA methylation β values at differentially methylated probes in TF binding motifs. We correlated *ERG* expression with average DNA methylation at 642 hyper-methylated and 24 hypo-methylated probes with overlapping *ERG* ChIP-Seq binding peaks (GSM353647). Due to the unavailability of ChIP-Seq peaks for the remaining TF hubs, we considered differentially methylated probes in promoters and enhancers with a TF binding motif. All the TF binding targets in our prostate network are enriched for TF motifs.

For this analysis, we used level 3 HM450 PRAD DNA methylation data for 333 TCGA prostate samples and log2-transformed level 3 PRAD TCGA RNA-Seq RSEM data. Three out of six TF hubs showed significant correlations between TF expression and DNA methylation at differentially methylated probes within TF binding motifs (*ERG*: rho = -0.173, *p* value = 0.00149; *POU2F2*: rho = -0.277, *p* value = 3.01 × 10^-7^; *SPI1*: rho = -0.14, *p* value = 0.010). The differential expression of TFs with more binding sites should have a larger impact in the network by causing methylation changes at more sites, consistent with our observation of significant correlation between TF hub expression (*ERG*, *POU2F2*, and *SPI1*) and DNA methylation at binding sites. As a reference, we do not observe significant correlation between expression of non-TF hubs (10% of the lowest out-degree nodes) and DNA methylation at differentially methylated probes within TF binding motifs.

### RWPE1 cell line preparation

We obtained RWPE1 cells from ATCC and maintained them as per the manufacturer’s protocol. The RWPE1-ERG isogenic cell line with overexpressed *ERG* (the common isoform is based on *TMPRSS2*-*ERG* fusion) and the RWPE1-GFP cell line have been previously described [97, 118]. We prepared genomic DNA from RWPE1-GFP or RWPE1-ERG using standard phenol chloroform extraction followed by ethanol preparation and suspension into 30 μl of 10 mM Tris pH 8.0. The genomic DNA used in the EpiTYPER MassARRAY assay was collected from the RWPE1-GFP/ERG cells using a system of purification through a column (NucleoSpin Tissue kit, Macherey Nagel, Bethlehem, PA).

### Sample preparation for ERRBS

Sample preparation was performed at Weill Cornell Medicine Epigenomics Core as previously described [94, 95]. ERRBS is a method developed to prepare DNA for base-pair resolution methylation sequencing analysis based on a restriction enzyme to enrich for CpG fragments [95]. This method is a modification of the original RRBS protocol described by [119] resulting in a 2× increase of CpG detection and coverage. Briefly, the sample preparation includes the following steps: (1) MspI enzyme digestion; (2) end repair of digested DNA; (3) adenylation; (4) adenylated DNA fragments are ligated with pre-annealed 5-methylcytosine-containing Illumina adapters; (5) library fragments of 150 to 400 bp are gel-isolated from a 1.5% agarose gel (using low-range ultra-agarose from Bio-Rad, Des Plaines, IL); (6) bisulfite conversion was performed using the EZ DNA Methylation Kit (Zymo Research, Irvine, CA) per the manufacturer’s recommendation with the following changes: (i) incubation after CT conversion was conducted in a thermocycler (Eppendorf, Hauppauge, NY) with the following conditions: 30 seconds at 95 °C followed by 15 minutes at 50 °C for 55 cycles, and (ii) product elution into 40 μl nuclease-free water; (7) polymerase chain reaction (PCR) amplification for each library was prepared with FastStart High Fidelity DNA Polymerase (Roche, Indianapolis, IN) and 0.5 μM each of the Illumina PCR primers PE1.0 and 2.0. The thermocycler conditions were 5 minutes at 94 °C, 18 cycles of 20 seconds at 94 °C, 30 seconds at 65 °C, 1 minute at 72 °C, followed by 3 minutes at 72 °C. PCR products were isolated using Agencourt AMPure XP beads per the manufacturer’s recommended protocol (Agencourt). All amplified libraries underwent quality control, which involves use of a Qubit 1.0 fluorometer and Quant-iT dsDNA HS Assay Kit (Invitrogen, Grand Island, NY) for quantitation and bioanalyzer visualization (Agilent 2100 Bioanalyzer; Agilent, Santa Clara, CA).

### EpiTYPER MassARRAY system

Sample preparation was performed at Weill Cornell Medicine Epigenomics Core as described in the previous section. Briefly, the gDNA samples are treated with bisulfite to convert any non-methylated cytosine residues into uracil. The targeted sequences are then amplified by PCR, preserving the bisulfite-induced sequence changes. In vitro transcription is performed and the resulting RNA transcripts are specifically cleaved at uracil residues. The resulting fragments differ in size and mass, depending on the sequence changes generated through bisulfite treatment. The EpiTYPER reaction products are dispensed onto a SpectroCHIP array and read by a MALDI-TOF mass spectrometer for data acquisition. The primers used to target the 17 sequences are listed in Additional file 4: Table S12.

### Computational approach for ERRBS analysis

The bisulfite-treated reads were aligned and methylation calls were made as previously described [95]. The bisulfite reads were aligned to the bisulfite converted hg19 reference genome using Bismark [120]. We analyzed the ERRBS data for both cell lines using the methylKit R package [121]. methylKit analyzes and characterizes genome-wide cytosine profiles from high-throughput methylation experiments. It reads DNA methylation information from text files and performs operations such as differential methylation analysis, sample clustering, annotation, and DNA methylation visualization [121]. We used ERRBS output files for RWPE1-ERG and RWPE1-GFP, which contained information about chromosome, base, strand, coverage, cytosine frequency (freqC), and thymine frequency (freqT). We used the calculateDiffMeth() function to find differentially methylated CpGs. The calculateDiffMeth() function uses Fisher’s exact test to compare the fraction of methylated C’s in test vs. control. methylKit uses the sliding linear model (SLIM) method to correct *p* values and report *q* values [121]. A CpG is defined to be differentially methylated if the percentage methylation difference between test and control is larger than 25% and the *q* value < 0.01. We used this criterion to call hyper- and hypo-methylated regions in the RWPE1-ERG cell line with respect to RWPE1-GFP (Additional file 1: Figure S11).

We compared DNA methylation in 333 TCGA prostate tumor samples with the DNA methylation for the RWPE1-ERG cell line and observed a significant correlation (Additional file 1: Figure S12).

## Additional files

Additional file 1: This file contains **Figures S1**–**S12** and Supplementary text. (PDF 11000 kb)

Additional file 2: Prostate regulatory network. This file contains TF–target gene edges in the prostate regulatory network. (TXT 14236 kb)

Additional file 3: This file includes **Tables S1**–**S4**. (XLSX 47 kb)

Additional file 4: This file includes **Tables S5**–**S12**. (XLSX 2964 kb)
