# Supplementary material for: Identification of novel prostate cancer drivers using RegNetDriver: a framework for integration of genetic and epigenetic alterations with tissue-specific regulatory network
Source: Genome Biol. 2017 Jul 27;18:141. doi: 10.1186/s13059-017-1266-3 (PMC5530464; doi:10.1186/s13059-017-1266-3)
Supplement: Supplementary file 1 — This file contains Figures S1–S12 and Supplementary text. (PDF 11000 kb) [file 13059_2017_1266_MOESM1_ESM.pdf]

Supplementary Figures and Text

Identification of novel prostate cancer drivers using RegNetDriver: a framework for integration of genetic and epigenetic alterations with tissue-specific regulatory network

Priyanka Dhingra[1,2], Alexander Martinez-Fundichely[1,2], Adeline Berger[3], Franklin W. Huang[4,5,6], Andre Neil Forbes[1,2], Eric Minwei Liu[1,2], Deli Liu[1,7], Andrea Sboner[2,3,8], Pablo Tamayo[6,9,10], David S. Rickman[3,8,11,*], Mark A. Rubin[3,8,11] and Ekta Khurana[1,2,8,11,*]

*Correspondence: ekk2003@med.cornell.edu, dsr2005@med.cornell.edu

Table of Contents

Figure S1: Distribution of target genes regulated by active prostate enhancers, and (b) distribution of prostate active enhancers associated with a target gene. 3

Figure S2: Receiver operator characteristic curves (ROC) and Precision-recall curves (PRC) for TF-target gene edges in RegNetDriver and Marbach *et al.* prostate network. 4-7

Figure S3: Area under receiver operator characteristic curve (AUROC) and precision-recall curve (AUPRC) for TF-promoter and TF-enhancer edges in RegNetDriver and Marbach. 8

Figure S4: Network Topology. 9

Figure S5: *FAS* mRNA expression in tumor samples with deletion vs. without deletion and tumor samples with hyper-methylated promoter vs. without hyper-methylation 10

Figure S6: Effect of genetic and epigenetic alterations on expression of *FAM3B* and *TNFSF13*. 11

Figure S7: Circos plot showing TF-target gene interaction edges for all the six TF hubs altered by SVs. 12

Figure S8: Expression difference between TCGA samples with and without TF hub deletions. 13

Figure S9: Correlation between TF hub expression and DNA methylation (β) at differentially methylated probes in TF binding motifs for tumor samples. 14

Figure S10: DNA methylation (β) values for 105,720 hyper-methylated and 120,957 hypo-methylated CpGs in RWPE1-ERG and RWPE1-GFP cell line. 15

Figure S11: Genomic locations of differentially methylated regions (DMRs) in RWPE1-ERG cell line with respect to control. 16

Figure S12: DNA methylation beta values for 66,301 probes from ERRBS experiment and TCGA tumor samples. 17

Supplementary text: Marbach *et al*. prostate epithelial network 18

Supplementary text: Role of DHS and motif information in network performance 18

Supplementary text: Structural Variants 18-19

(a)

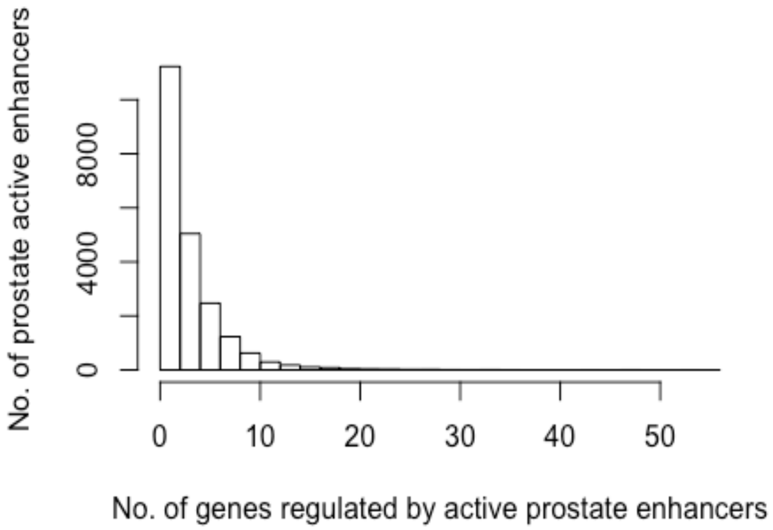

(b)

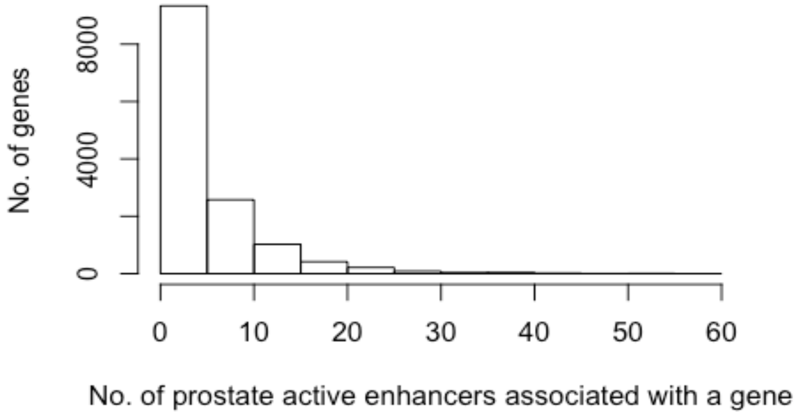

Figure S1 (a) Distribution of target genes regulated by active prostate enhancers, and (b) distribution of prostate active enhancers associated with a target gene.

(a)

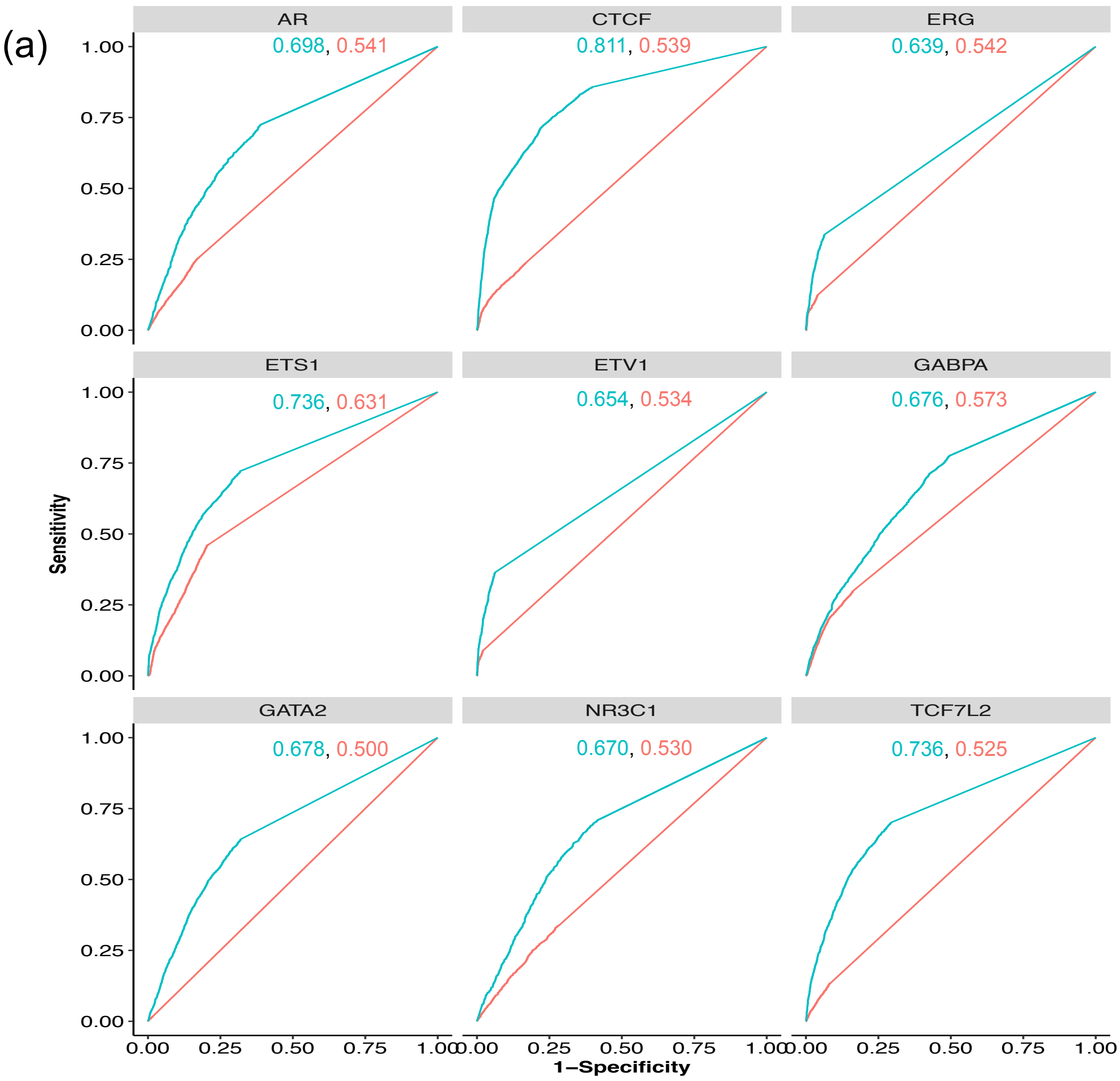
AR
0.698, 0.541
CTCF
0.811, 0.539
ERG
0.639, 0.542
ETS1
0.736, 0.631
ETV1
0.654, 0.534
GABPA
0.676, 0.573
GATA2
0.678, 0.500
NR3C1
0.670, 0.530
TCF7L2
0.736, 0.525
Sensitivity
1−Specificity
0.00
0.25
0.50
0.75
1.00

(b)

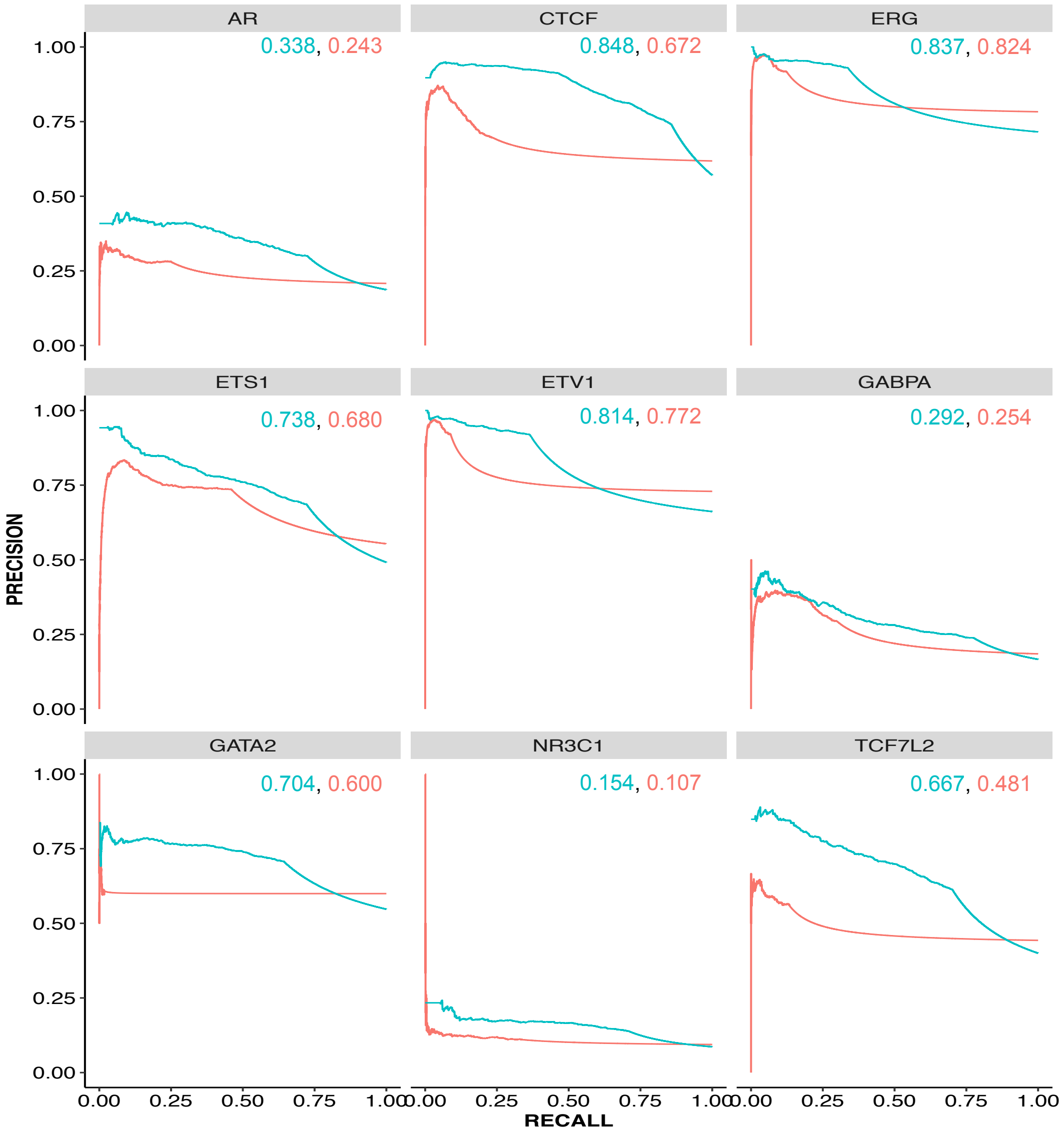
AR
CTCF
ERG
0.338, 0.243
0.848, 0.672
0.837, 0.824
ETS1
ETV1
GABPA
0.738, 0.680
0.814, 0.772
0.292, 0.254
GATA2
NR3C1
TCF7L2
0.704, 0.600
0.154, 0.107
0.667, 0.481
PRECISION
RECALL
0.00
0.25
0.50
0.75
1.00

(c)

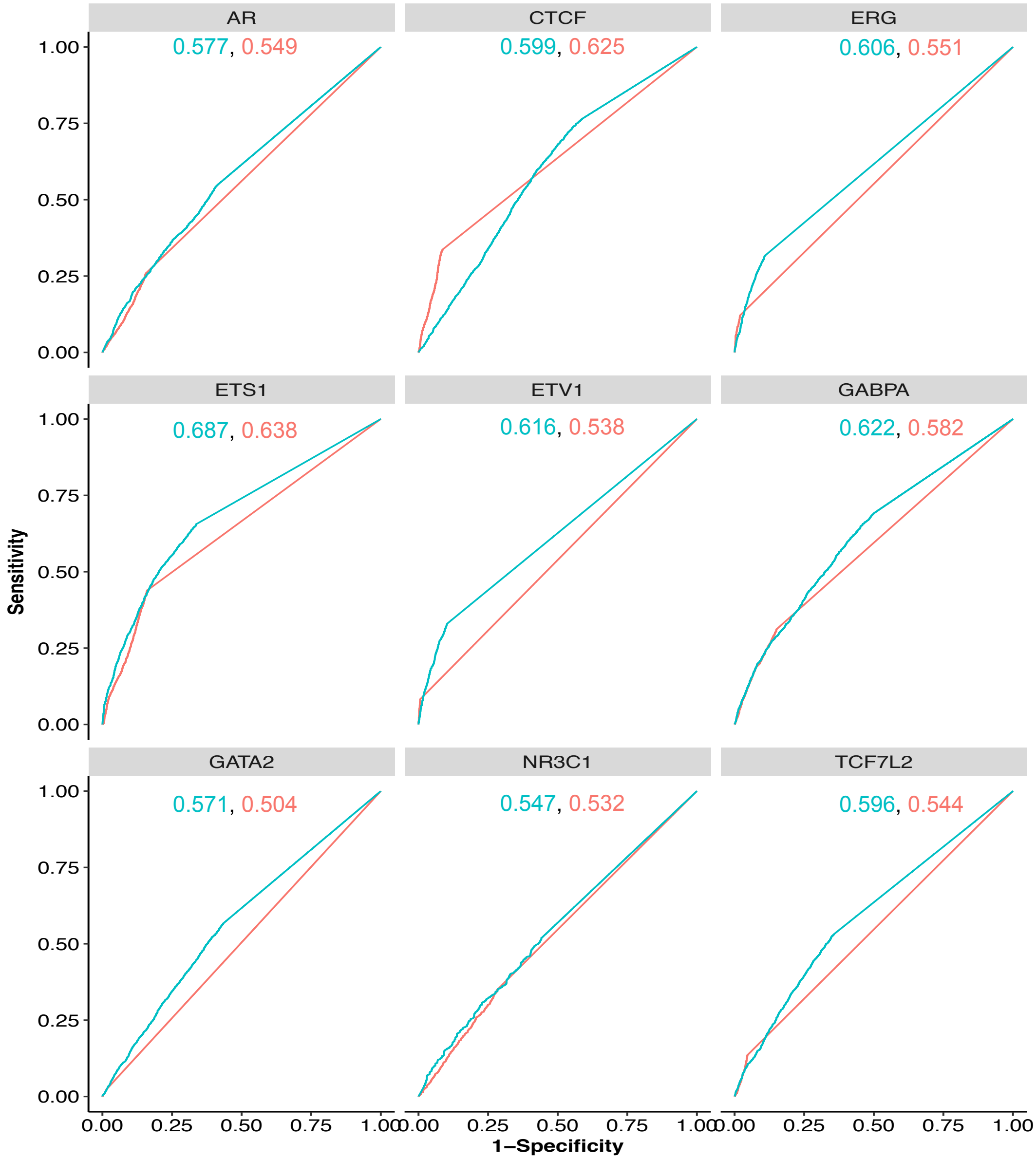
AR
0.577, 0.549
CTCF
0.599, 0.625
ERG
0.606, 0.551
ETS1
0.687, 0.638
ETV1
0.616, 0.538
GABPA
0.622, 0.582
GATA2
0.571, 0.504
NR3C1
0.547, 0.532
TCF7L2
0.596, 0.544
Sensitivity
1−Specificity
0.00
0.25
0.50
0.75
1.00

(d)

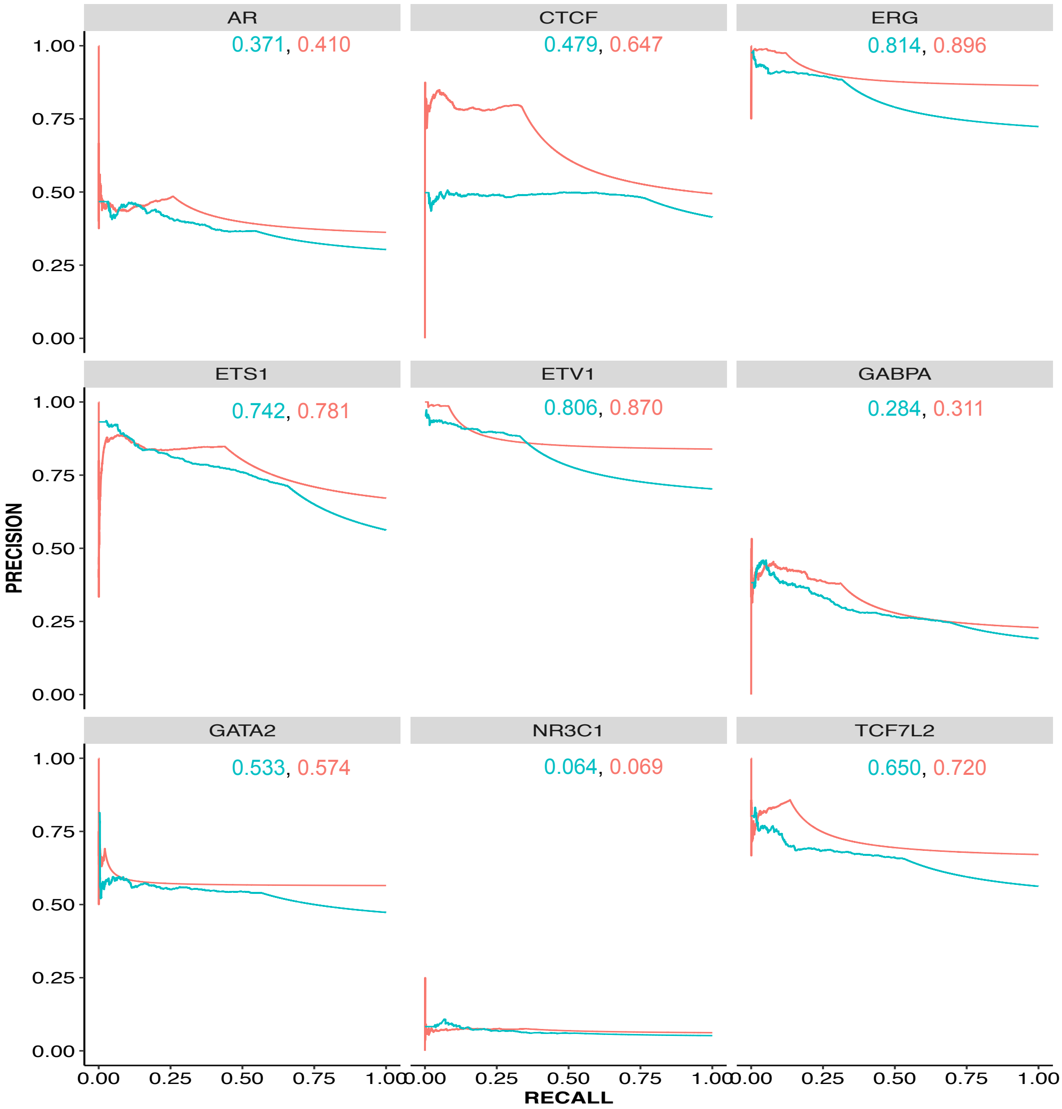

Figure S2 Receiver operator characteristic curves (ROC) and Precision-recall curves (PRC) for TF-target gene edges in RegNetDriver (green) and Marbach *et al*. (red) prostate network. (a and b) ROC and PRC for TF-target gene edges evaluated using ChIP-RegNetDriver genes. (c and d) ROC and PRC for TF-target gene edges in RegNetDriver and Marbach network evaluated using ChIP-Marbach targets. Insets in a and c represent AUROC for RegNetDriver (green) and Marbach (red) network. Insets in b and d show AUPRC for both the networks.

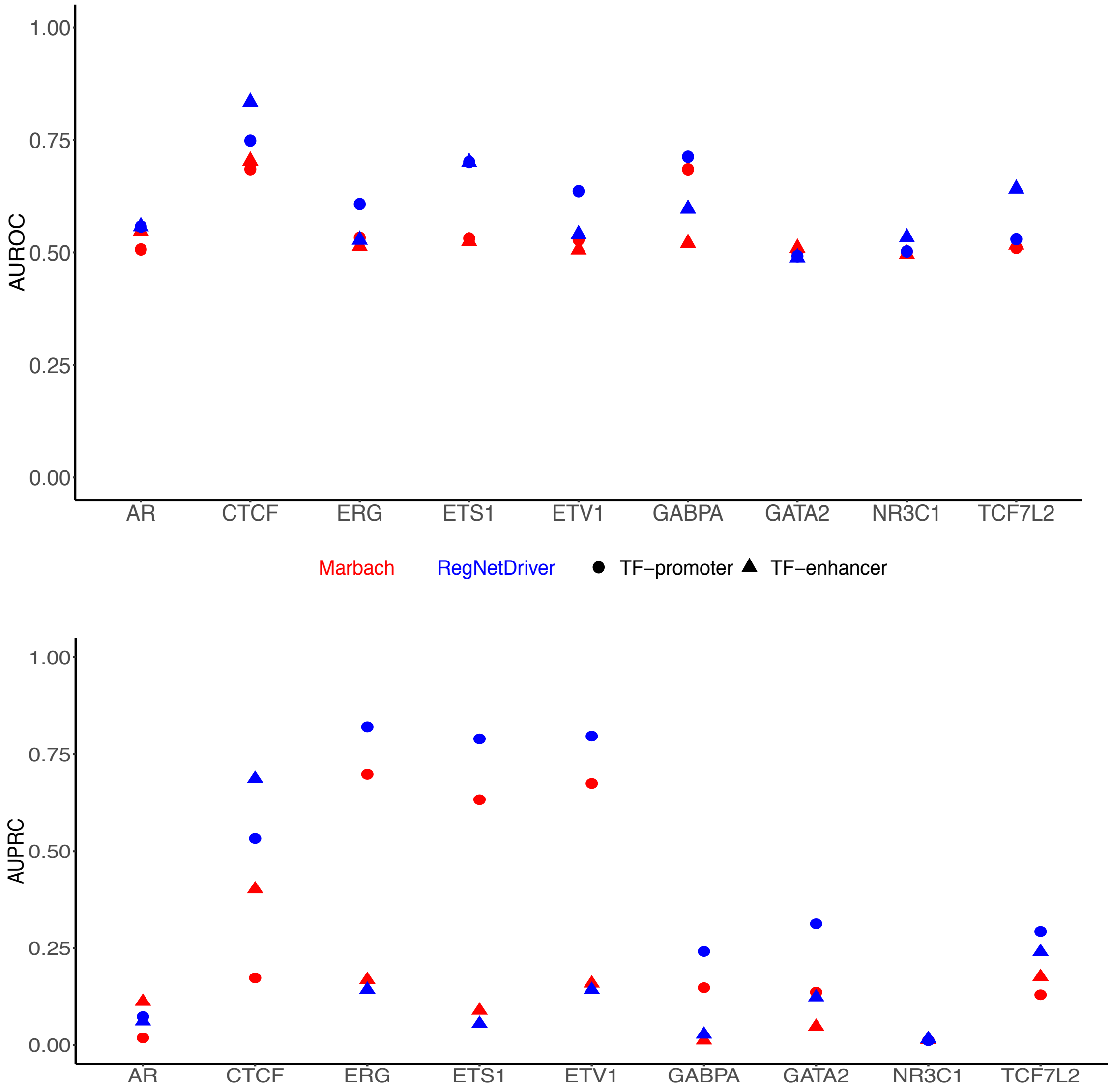

Figure S3 Area under receiver operator characteristic curve (AUROC) and precision-recall curve (AUPRC) for TF-promoter and TF-enhancer edges in RegNetDriver (blue) and Marbach (red). Circular data points represent AUROC and AUPRC for TF-promoter edges evaluated using TF-ChIP-Seq peaks and triangular data points show AUROC and AUPRC values for TF-enhancer edges.

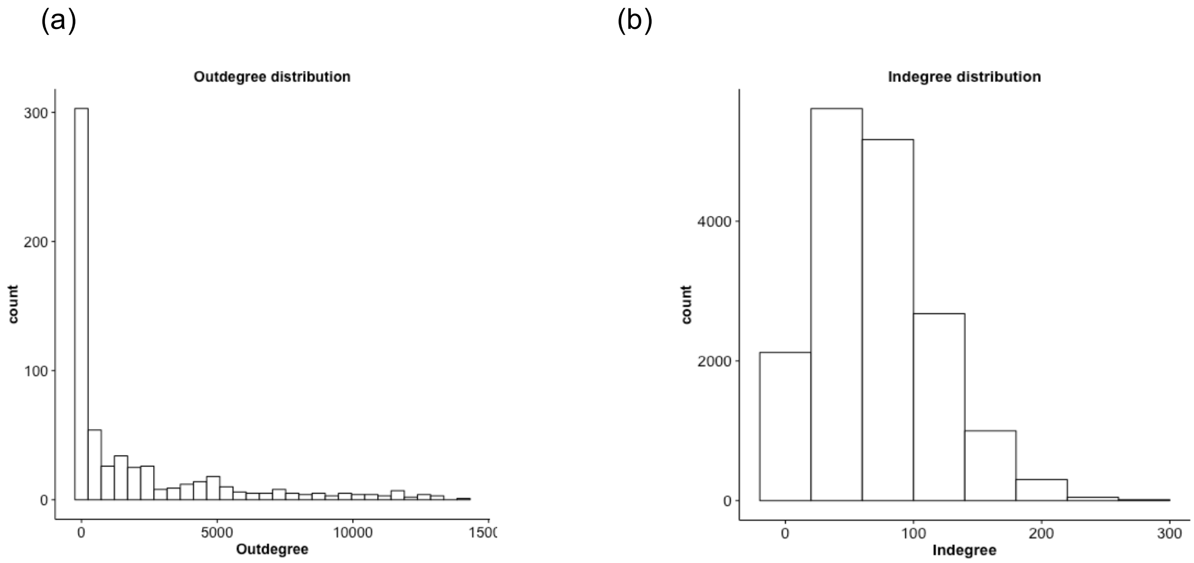

Figure S4 Network topology. (a) Out degree distribution of prostate regulatory network and (b) In degree distribution of prostate regulatory network.

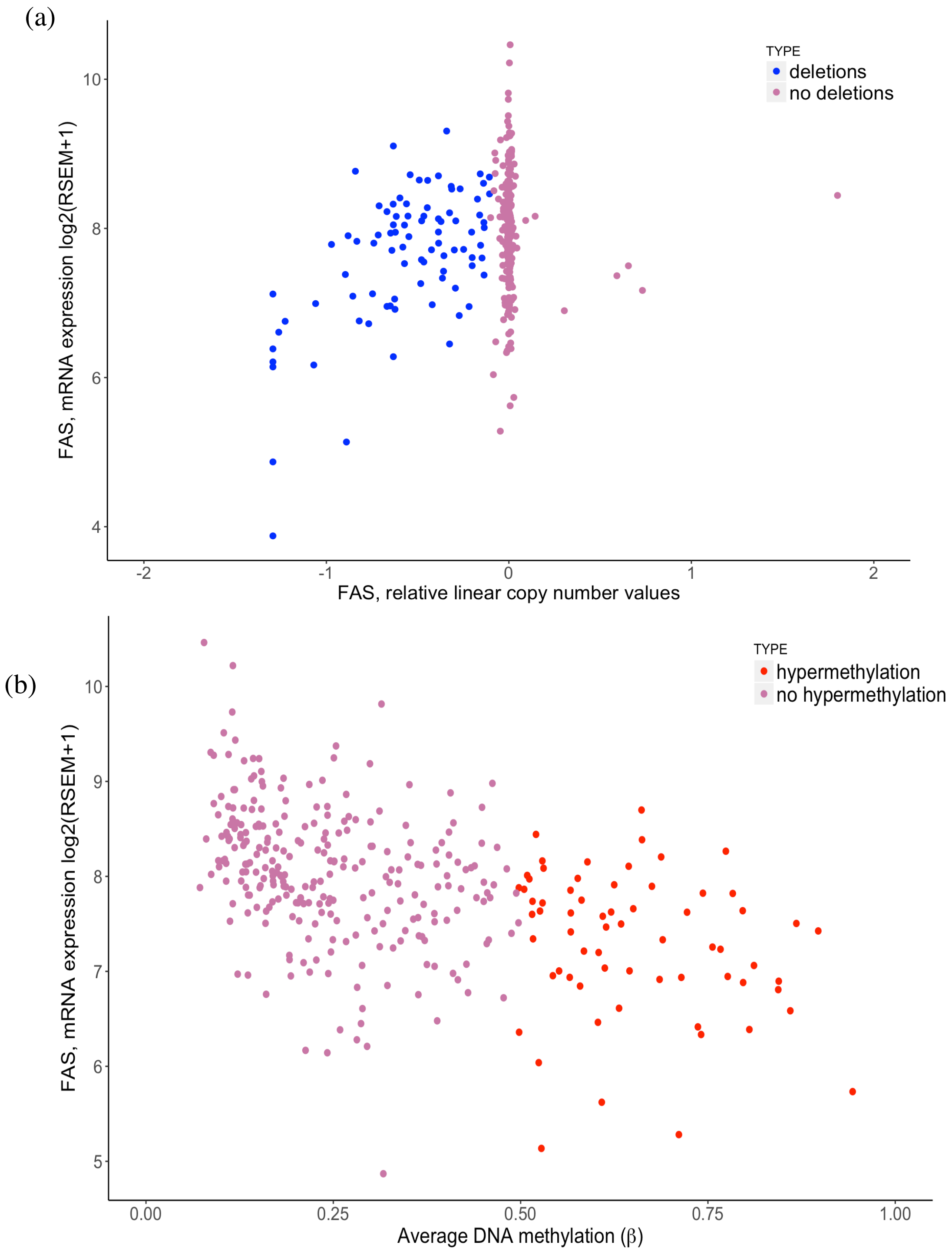

Figure S5 Scatter plots present change in *FAS* mRNA expression in tumor samples with deletion (blue) vs. without deletion (pink) and tumor samples with hyper-methylated promoter (red) vs. without hyper-methylation (pink)

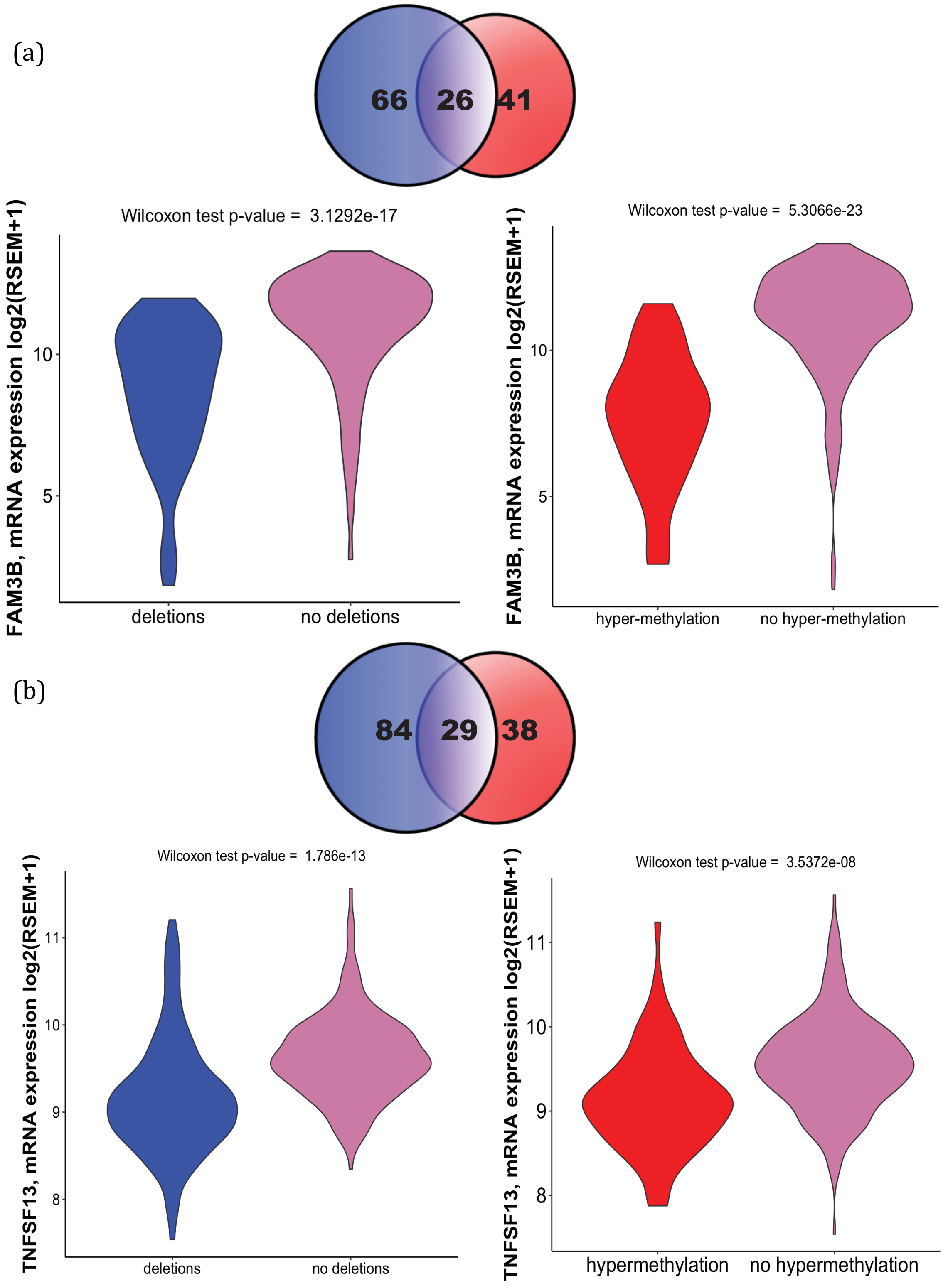

Figure S6 Effect of genetic and epigenetic alterations on expression of *FAM3B* and *TNFSF13*. (a) Venn diagram shows the number of samples affected by deletion (blue) and promoter hyper-methylation (red) and the violin plot represents the change in expression of *FAM3B* in tumor samples with deletion (n=96) and tumor samples with hyper-methylated promoters (n=67) and (b) Venn diagram shows the number of samples affected by deletion and promoter hyper-methylation and the violin plots represent the change in expression of *TNFSF13* in tumor samples with deletion (n=113) and tumor samples with hyper-methylated promoters (n=67).

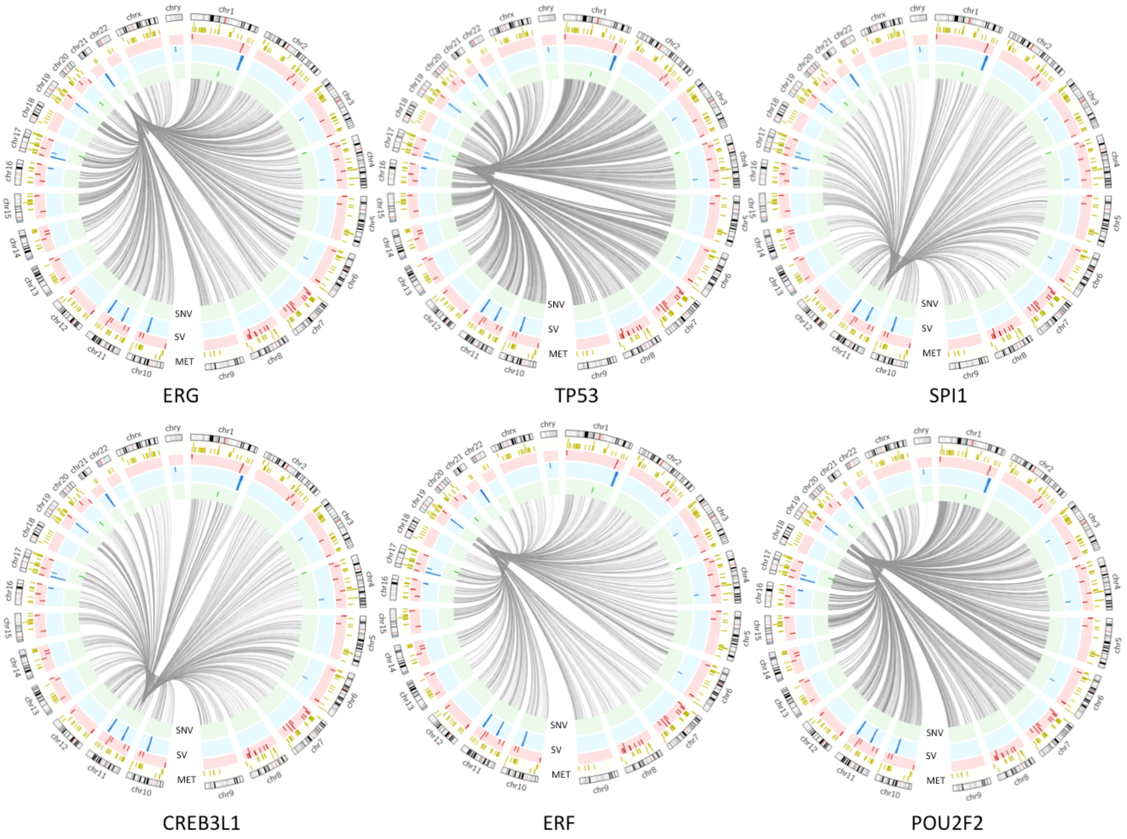

Figure S7 Circos plot showing TF-target gene interaction edges for all the six TF hubs altered by SVs. All the plots contain TF-target gene interactions in the center (shown in grey), genomic location of TF hub is shown in the outer ring, genes significantly altered by SNVs in coding sequence, promoters and enhancers are shown as green bars, genes significantly altered by SVs in coding sequence, promoters and enhancers are shown as blue bars, genes with hypo-methylated promoters and enhancer are shown in yellow, and genes with hyper-methylated promoters and enhancers are shown in red. Refer Figure 5 for more details.

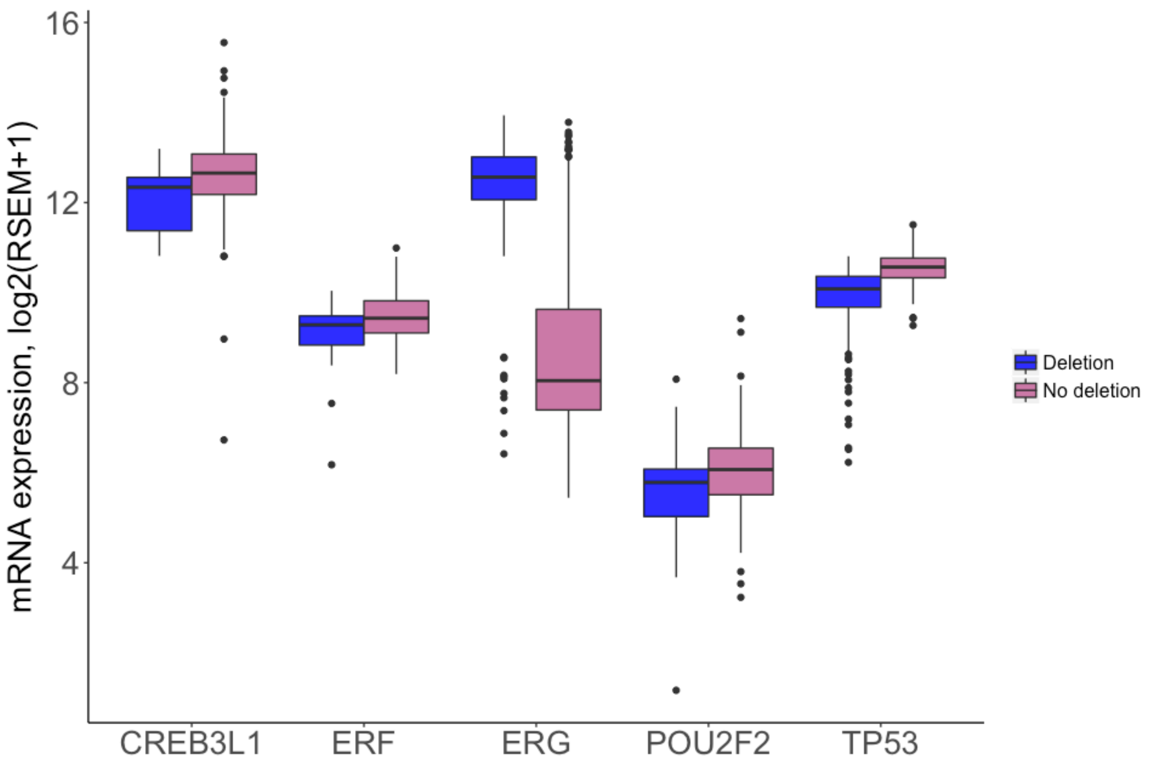

Figure S8 Expression difference between TCGA samples with and without TF hub deletions. Samples with deletions are in blue and samples without deletions are in pink. The significant increase in ERG expression is due to interstitial deletions between *TMPRSS2* and *ERG*. Other TF hubs showed a significant decrease in expression for samples with deletions (Wilcoxon test p-value < 0.05).

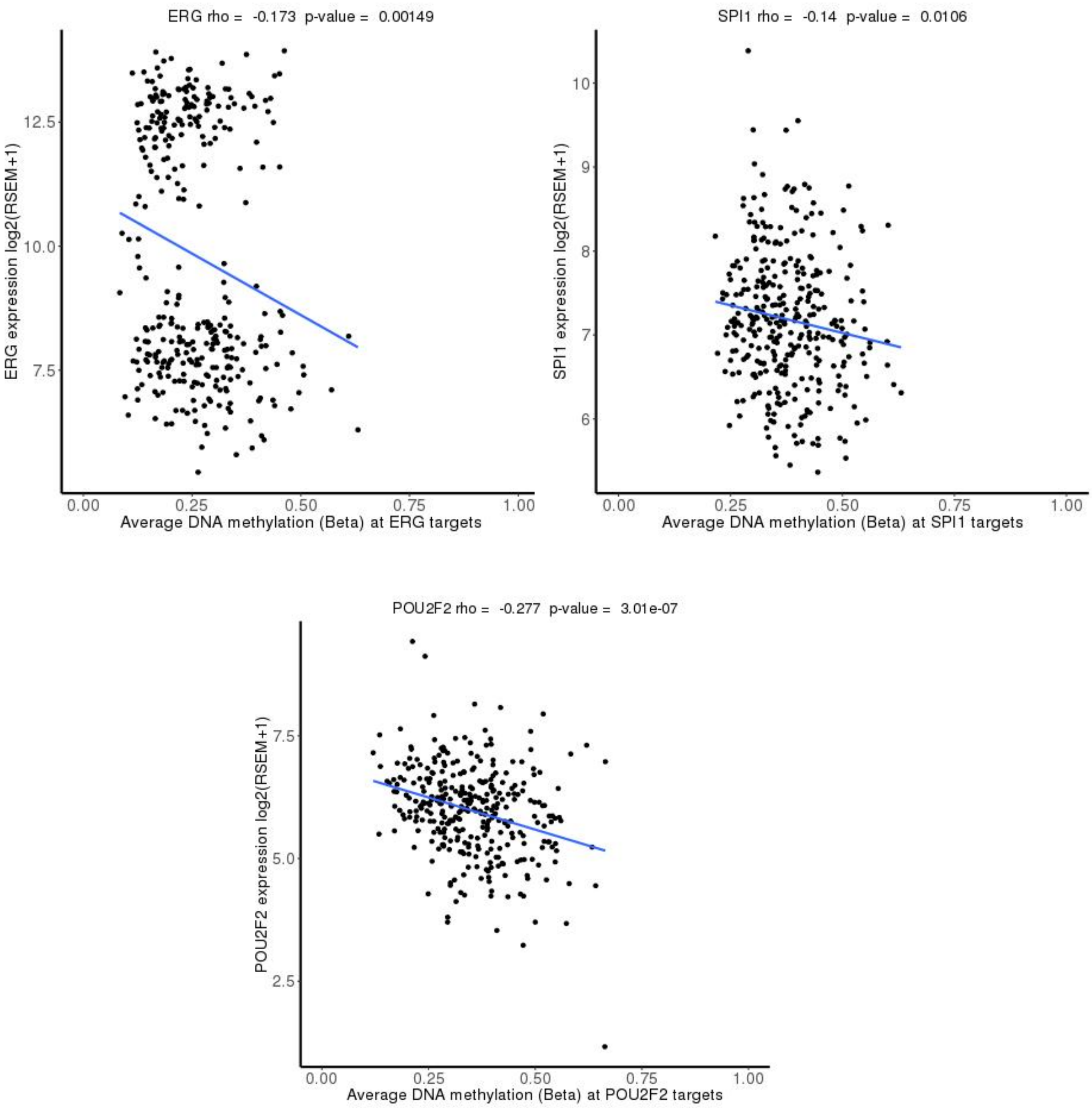

Figure S9 Correlation between TF hub expression and DNA methylation (β) at differentially methylated probes in TF binding motifs for tumor samples (n=333). A significant association (pvalue <0.05) is observed between TF hub expression and average DNA methylation across all the differentially methylated probes with TF binding sites.

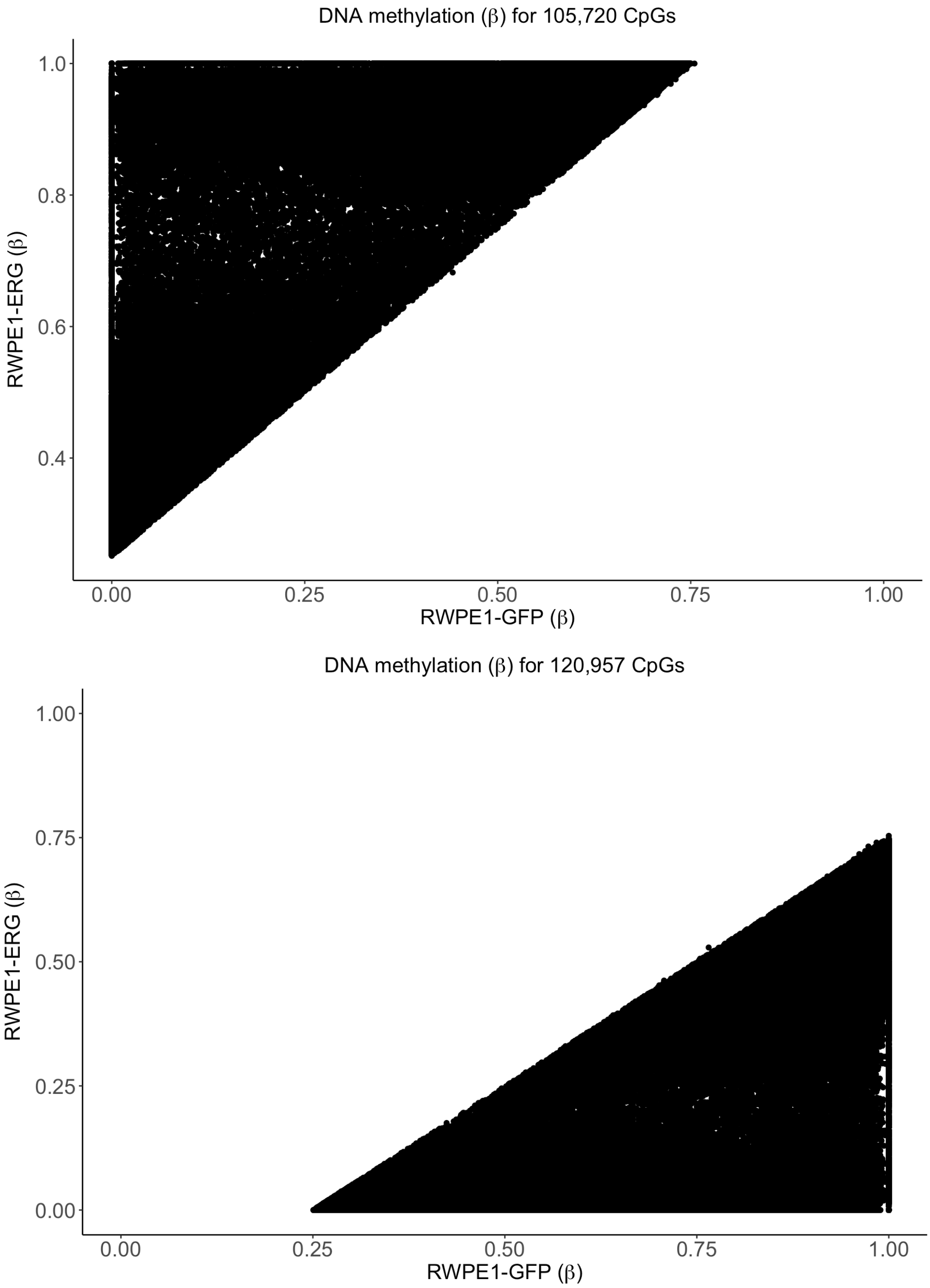

Figure S10 Scatter plots showing DNA methylation (β) values for 105,720 hyper-methylated and 120,957 hypo-methylated CpGs in RWPE1-ERG and RWPE1-GFP cell line. A CpG is defined as differentially methylated if percent methylation difference between test and control is larger than 25% and qvalue <0.01.

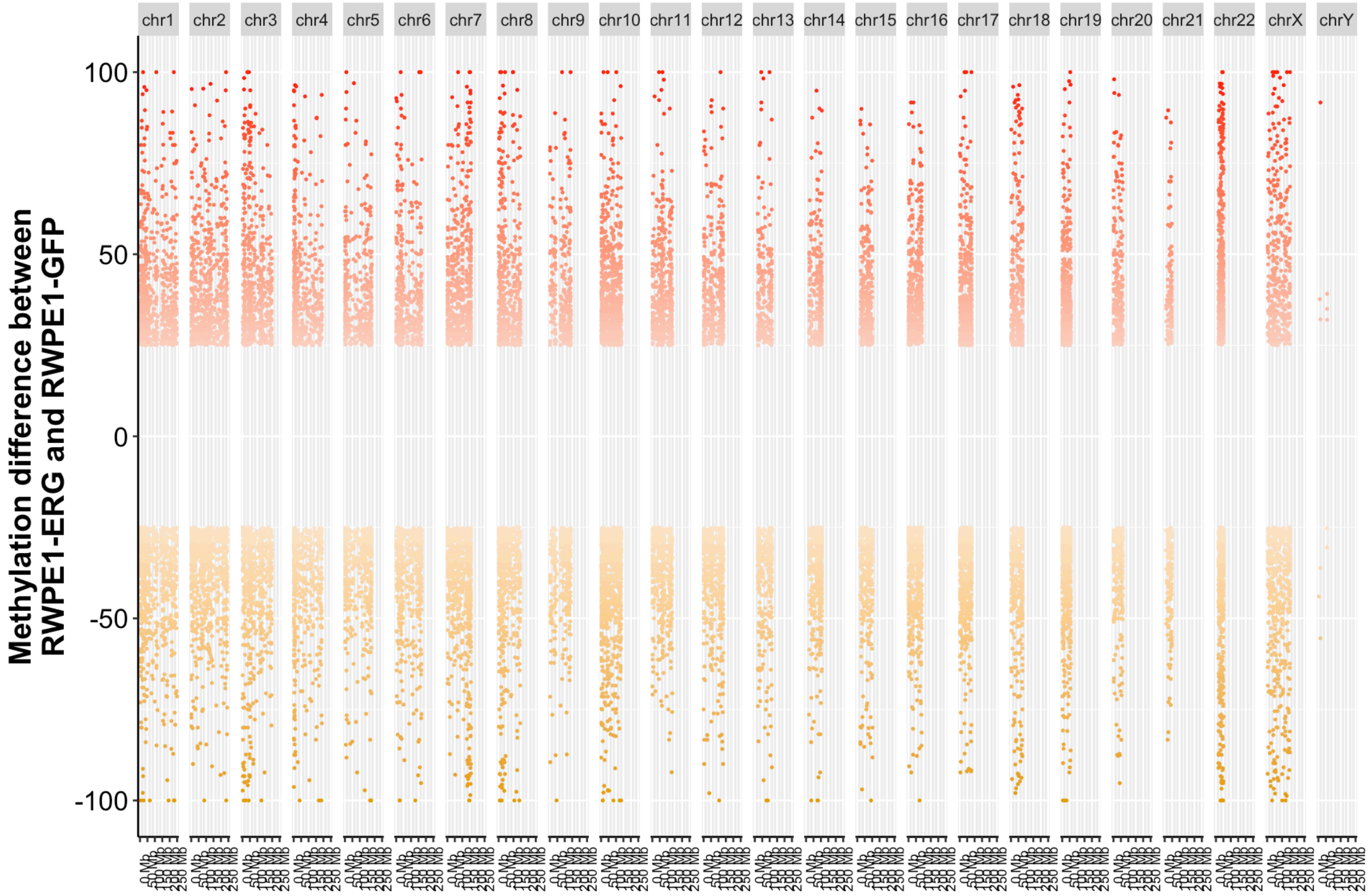

Figure S11 Genomic locations of differentially methylated regions (DMRs) in RWPE1-ERG cell line with respect to control. Each data point represents a DMR of 1 KB. Red dots represent hyper-methylated regions and yellow dots represent hypo-methylated regions. Methylation difference between RWPE1-ERG and RWPE1-GFP is shown with a gradient color scheme.

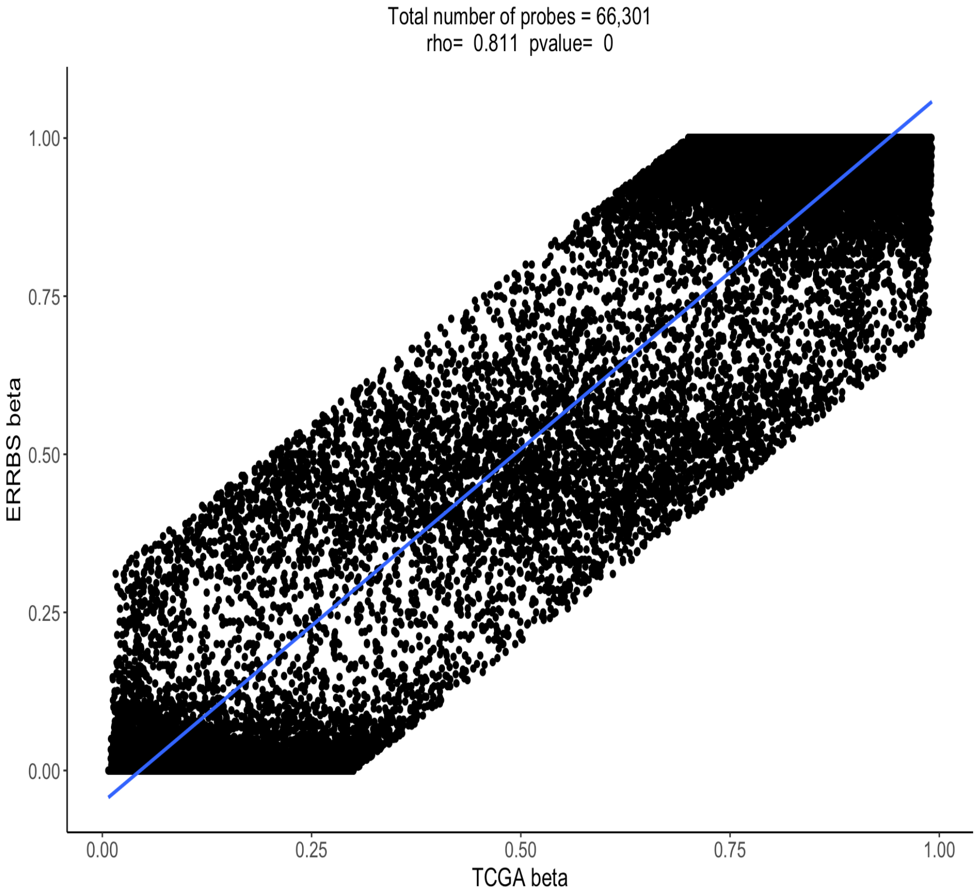

Figure S12 DNA methylation beta values for 66,301 probes from ERRBS experiment and TCGA tumor samples. We compared DNA methylation in 333 TCGA prostate tumor samples with the DNA methylation for RWPE1-ERG cell line. For 79,387 probes we had DNA methylation beta values of RWPE1-ERG cell line from ERRBS experiment and TCGA prostate samples. On comparing beta value for these probes, we observed a significant correlation of 0.8 (rho=0.8, p=2.2*10-16) where β=M/(M+U), where M= number of methylated Cs and U=number of unmethylated Cs. Overall, 84% probes (66,301 probes) showed methylation difference of $\leq$ 0.3 between TCGA and ERRBS (| βtcga-βerrbs |<=0.3).

Supplementary text

Marbach *et al* prostate epithelial network: We downloaded the prostate epithelial network and supplementary data from http://regulatorycircuits.org/. Following is a list of files used for network evaluation:

(a) Prostate epithelial network- prostate_epithelial_cells.txt.gz. (Available at: http://www2.unil.ch/cbg/regulatorycircuits/FANTOM5_individual_networks.tar)

(b) Tissue agnostic promoter-gene annotations- promoter---transcript.prec90.txt
(c) Tissue agnostic enhancer-gene annotations- enhancer---transcript.prec90.txt
(d) TF motifs enriched in CAGE-defined promoters- tf---promoter.prec90.bed
(e) TF motifs enriched in CAGE-defined enhancers- tf---enhancer.prec90.bed
(f) CAGE-defined promoters- promoter_expr.rank.prec90.txt.gz
(g) CAGE-defined enhancers- enhancer_expr.rank.txt.

Files b-g are available inside supplementary data folder http://www2.unil.ch/cbg/regulatorycircuits/Supplementary_data.zip)

Role of DHS and motif information in network performance: We tried to assess the individual contributions of DHS and motif information in network performance. For this analysis, we constructed a prostate regulatory network using DHS information only and considered all the genes associated with active prostate promoters and enhancers as AR DHS targets (17,023 target genes). We compared AR DHS targets with ChIP-Seq peaks (GSM1358399). We compared 17,023 AR DHS target genes with 3,759 ChIP-based AR target genes. There are 3,713 true positive, 13,310 false positive and 46 false negative edges. We obtained a sensitivity (true positive rate) of 0.9877627, specificity (true negative rate) of 0.1861816 and precision of 0.2181167. Thus, DHS-only network shows low specificity and precision and highlights the importance of motif information in network construction. DHS are highly predictive of cell-type specific regulatory regions and contribute to network prediction sensitivity, while motif information in our framework increases specificity and precision by reducing network complexity in terms of number of edges. Together they assist in reliably recapitulating global transcriptional regulatory interactions.

Structural variants from 124 prostate tumor samples, ICGC: Delly (v0.5.5) was used to call genomic rearrangements in ICGC dataset at minimum median mapping quality of 20 and paired-end-cutoff of five. The resulting somatic variants were filtered using a consolidated list of SVs from 124 normal samples. Further detail about the whole-genome sequencing data analysis in the ICGC dataset is given in Ref. 47. SV data can be downloaded from https://dcc.icgc.org/projects/PRAD-CA.

Structural variants from 64 prostate tumor samples, Baca *et al* and Berger *et al*: Structural variants in 64 prostate tumor samples were called using dRanger. The final somatic datasets was filtered against a panel of 176 normal tissue genomes. Further details are provided in ref 9 and 5. The SV datasets for *Baca et al*. and Berger *et al*. can be downloaded from www.ncbi.nlm.nih.gov/pmc/articles/PMC3690918/bin/NIHMS472000-supplement-03.xls (Table S3C) and http://www.nature.com/nature/journal/v470/n7333/extref/nature09744-s2.xlsx (Table S5).

In total, we used 3,176 deletions, 1,464 inversions, 1,112 duplications, 1,732 translocations and 2,869 long-range interactions from 188 prostate tumor samples to run FSig-SV algorithm.
